# Volatiles of pathogenic and non-pathogenic soil-borne fungi affect plant development and resistance to insects

**DOI:** 10.1007/s00442-019-04433-w

**Published:** 2019-06-15

**Authors:** Kay Moisan, Viviane Cordovez, Els M. van de Zande, Jos M. Raaijmakers, Marcel Dicke, Dani Lucas-Barbosa

**Affiliations:** 1 Laboratory of Entomology, Wageningen University and Research, Wageningen, The Netherlands; 2 Department of Microbial Ecology, Netherlands Institute of Ecology, Wageningen, The Netherlands; 3 Institute of Biology, Leiden University, Leiden, The Netherlands; 4 Present Address: Bio-communication and Ecology, ETH Zürich, Zurich, Switzerland

**Keywords:** *Arabidopsis thaliana*, Fungal volatiles, Plant development, Plant pathogens, Plant resistance

## Abstract

Plants are ubiquitously exposed to a wide diversity of (micro)organisms, including mutualists and antagonists. Prior to direct contact, plants can perceive microbial organic and inorganic volatile compounds (hereafter: volatiles) from a distance that, in turn, may affect plant development and resistance. To date, however, the specificity of plant responses to volatiles emitted by pathogenic and non-pathogenic fungi and the ecological consequences of such responses remain largely elusive. We investigated whether *Arabidopsis thaliana* plants can differentiate between volatiles of pathogenic and non-pathogenic soil-borne fungi. We profiled volatile organic compounds (VOCs) and measured CO_2_ emission of 11 fungi. We assessed the main effects of fungal volatiles on plant development and insect resistance. Despite distinct differences in VOC profiles between the pathogenic and non-pathogenic fungi, plants did not discriminate, based on plant phenotypic responses, between pathogenic and non-pathogenic fungi. Overall, plant growth was promoted and flowering was accelerated upon exposure to fungal volatiles, irrespectively of fungal CO_2_ emission levels. In addition, plants became significantly more susceptible to a generalist insect leaf-chewing herbivore upon exposure to the volatiles of some of the fungi, demonstrating that a prior fungal volatile exposure can negatively affect plant resistance. These data indicate that plant development and resistance can be modulated in response to exposure to fungal volatiles.

**Electronic supplementary material:**

The online version of this article (10.1007/s00442-019-04433-w) contains supplementary material, which is available to authorized users.

## Introduction

Plants are exposed to diverse communities of insects and microorganisms, ranging from beneficial organisms, such as natural enemies of herbivores and plant growth-promoting rhizobacteria, to deleterious organisms such as pests and pathogens (Raaijmakers et al. 2009; Bardgett and van der Putten 2014; Stam et al. 2014). To cope with antagonists and maximise mutualistic interactions, plants have evolved specific mechanisms to recognize elicitors from pathogens and insect herbivores, also referred to as microbe-associated molecular patterns and herbivore-associated molecular patterns, respectively.

Upon recognition of pathogenic microbes colonising the roots, local and systemic responses are induced in the plant which may affect plant development and resistance (Chagas et al. 2018). Recognition of pathogens by plants usually leads to programmed cell death at the site of infection, known as the hypersensitive response, and accumulation of reactive oxygen species, preventing further infection of the plant tissues (Wojtaszek 1997; Coll et al. 2011). Upon infection, plants may also reallocate their resources, which, in turn, affect growth and accelerate reproductive development (Korves and Bergelson 2003; Berger et al. 2007). Also non-pathogenic microorganisms that colonise roots can alter plant development and induce resistance against a range of biotic stresses (Bent 2006; Pineda et al. 2010; Pieterse et al. 2014) and abiotic stresses (van Wees et al. 2008). Furthermore, some non-pathogenic microorganisms can promote plant growth through facilitation of nutrient uptake or hormone production, and via symbiotic interactions such as nitrogen fixation (van Loon 2007; Bhattacharyya and Jha 2012). Plants colonised by these beneficial soil microorganisms may also accelerate flowering, thus, promoting their reproductive output (Koide and Dickie 2002; Wolfe et al. 2005). Hence, both pathogens and non-pathogenic microorganisms can affect plant fitness.

Remarkably, plants can also respond to microorganisms from a distance via the perception of microbial volatiles, encompassing volatile organic compounds (VOCs) and inorganic volatile compounds such as CO_2_ and NO (Schmidt et al. 2015). Already at the seed stage, microbial volatiles can affect plant development and delay seed germination (Ogura et al. 2000; Hung et al. 2014). At later growth stages, exposure to microbial volatiles may affect flowering time positively or negatively (Xie et al. 2009; Sánchez-López et al. 2016). In response to exposure to microbial volatiles, plant growth can be promoted (Naznin et al. 2013; Kanchiswamy et al. 2015; Piechulla and Schnitzler 2016; Bailly and Weisskopf, 2017; Piechulla et al. 2017) or inhibited (Wenke et al. 2012; Lo Cantore et al. 2015; Lee et al. 2016). Interestingly, plant exposure to microbial volatiles can also enhance plant resistance to foliar pathogens (Farag et al. 2013; Naznin et al. 2014; Ryu et al. 2004; Sharifi and Ryu 2016). However, effects of microbial volatiles on plant resistance to herbivorous insects are overlooked and contrasting (Song and Ryu 2013; D’Alessandro et al. 2014; Aziz et al. 2016; Cordovez et al. 2017). Collectively, these studies suggest a degree of specificity in plant responses to microbial volatiles.

Perception of distinct volatile profiles by plants may be one of the mechanisms underlying specificity of plant responses to different microorganisms. Fungal species with different lifestyles, such as ectomycorrhiza, pathogens, and saprophytes, are, indeed, known to emit unique VOCs which can be used as additional biomarkers for phylogenetic delineation (Müller et al. 2013; Cordovez et al. 2015; Oliveira et al. 2015). Palma et al. (2018) recently proposed that profiling of the presence/absence of certain subsets of VOCs could be used to determine if a given microbial species is pathogenic to humans. Yet, it remains unclear whether plants can discriminate between volatiles of pathogenic and non-pathogenic fungi and respond accordingly (Bitas et al. 2015; Casarrubia et al. 2016; Sánchez-López et al. 2016; Cordovez et al. 2017; Tahir et al. 2017; Hernández-Calderón et al. 2018; Li et al. 2018). Here, we hypothesized that plants can distinguish between volatiles of pathogenic and non-pathogenic soil-borne fungi. Volatiles of pathogenic fungi may be perceived as a ‘warning’ of the presence of a potential antagonist, allowing plants to prepare for the attack, whereas volatiles of non-pathogenic fungi may be perceived as an information of a potential mutualist, prompting plants to facilitate direct contact. To test this hypothesis, we (1) selected multiple pathogenic and non-pathogenic soil-borne fungi, (2) analysed their VOC profiles and quantified their CO_2_ emission, and (3) compared the phenotypic responses of *Arabidopsis thaliana* seedlings upon exposure to these fungal volatiles. More specifically, we investigated if exposure of seedlings to fungal volatiles affects plant development and resistance to a generalist herbivorous leaf-chewing insect.

## Materials and methods

### Study system

The annual plant *Arabidopsis thaliana* L. (Brassicaceae) was used as model plant. Eleven soil-borne fungi (belonging to ten different species) were selected to investigate their volatile-mediated interactions with *A. thaliana.*

Fungi were selected on the basis of three criteria: (1) part of the fungal life cycle is saprophytic; (2) fungi were previously isolated from brassicaceous plants; and (3) fungi co-occur in regions where *A. thaliana* is naturally present (Table S1). We selected five fungi that are economically important pathogens of brassicaceous crops (Table S2, Fig. S1): *Verticillium longisporum* (Zhou et al., 2006); *Verticillium dahliae* (Fradin and Thomma, 2006); *Sclerotinia sclerotiorum* (Dickman and Mitra, 1992)*; Fusarium oxysporum* f. sp*. raphani* (Leeman et al., 1995), and *Rhizoctonia solani* (Pannecoucque and Höfte, 2009). The six other fungi are non-pathogenic to *A. thaliana*, being either rhizospheric fungi or endophytes: *Trichoderma viride* (Harman et al., 2004)*; Ulocladium atrum* (Junker et al., 2012)*; Chaetomium indicum* (Keim et al., 2014)*; Fusarium oxysporum* Fo47 (Alabouvette, 1999); *Phoma leveillei* (Junker et al., 2012), and *Mucor plumbeus* (Ishimoto et al., 2000).

We selected the leaf chewer *Mamestra brassicae* L. (Lepidoptera: Noctuidae) as a generalist herbivorous insect species. This lepidopteran species, known as the cabbage moth, is an important pest on a broad range of crops including cabbage plants (Ahuja et al. 2010).

### Culture of fungi, plants, and insects

*Arabidopsis thaliana* seeds (accession Columbia-0) were surface-sterilised by exposure to chlorine gas for 3 h in a desiccator (Cordovez et al. 2017) and stratified in the dark for 3–4 days at 4 °C. Six seeds were sown per 9 cm ø Petri dish containing half-strength Murashige–Skoog medium (Duchefa, The Netherlands) with vitamins and supplemented with 5% sucrose. The medium pH was set at 5.8. Petri dishes were sealed with plastic wrap (Darco Pack B.V., The Netherlands) and kept vertically in a climate cabinet (21 ± 1 °C; 180 µmol light m^−2^ s^−1^; 16:8 h, L:D; 70 ± 5% R.H.) for 1 week prior to the exposure to the fungal volatiles. One Petri dish was treated as one biological replicate.

All Petri dishes with fungi were initiated with a mycelial plug collected from a fungal culture, apart from *V. dahliae* and *V. longisporum*, for which the spores were used. For the collection of fungal volatiles, fungi were grown in 7 cm ø glass Petri dishes. For all bioassays in which plants were exposed to the fungal volatiles, fungi were grown in ø 3 cm plastic Petri dishes. All fungi were cultured on 1/5th strength Potato Dextrose Agar (1/5th PDA), prepared with 7.8 g of PDA (Oxoid) and 14 g of technical agar number 3 (Oxoid). The medium pH was set at 7. Petri dishes were incubated at 25 °C in the dark until fungi reached a diameter of 3 cm (Table S1). Thus, the fungi *T. viride* and *M. plumbeus* were incubated for 4 days; *V. longisporum, V. dahliae, R. solani, U. atrum, F. oxysporum 47,* and *F. oxysporum* f.sp. *raphani* were incubated for 7 days; *C. indicum, S. sclerotiorum,* and *P. leveillei* were incubated for 10 days at 25 °C. Fungi with different incubation times were grown with their respective controls, i.e., medium alone incubated for 4, 7 or 10 days.

Caterpillars of *M. brassicae* were reared on *Brassica oleracea* L. var. *gemmifera* cv. Cyrus (Brussels sprouts) plants. The adults were kept in glass containers and fed with a sucrose solution (10%) in a climate chamber (22 ± 2 °C; 16:8 h, L:D; 50 ± 5% R.H.). Freshly laid eggs were used in the experiments.

### Collection and analysis of fungal volatile organic compounds

To profile VOCs emitted by pathogenic and non-pathogenic fungi, we collected VOCs from the headspace of the 11 fungi. As a control, we collected and determined VOCs emitted by the medium used to culture the different fungi (1/5th PDA). Fungal VOCs were collected for 2 h using a dynamic headspace set-up and each fungus was replicated six times. VOCs were collected from fungi previously grown in the dark at 25 °C for 4, 7, or 10 days (Table S1), in glass Petri dishes (7 cm ø) containing 1/5th PDA medium. Each Petri dish was placed individually inside a 0.5 l glass jar (previously autoclaved) with an inlet and an outlet. Synthetic air was flushed into the jar at a flow rate of 300 ml min^−1^ via a Teflon tube inserted through the inlet in the jar lid. Fungal VOCs were collected in a tube filled with 90 mg Tenax TA 25/30 mesh (Grace-Alltech, Germany) connected directly to the outlet on the lid of the glass jar, and air was sucked out at a flow rate of 200 ml min^−1^ (224-PCMTX8, air-sampling pump Deluxe, Dorset, UK; equipped with an inlet protection filter) for 2 h in a greenhouse compartment (25 ± 2 °C, 16:8 h, L:D, 60 ± 5% R.H.). Fungal dry weight was determined and used to normalise the peak area of each compound. For this, mycelium-containing agar was cut into pieces and transferred to a glass beaker with demineralised water (Garbeva et al. 2014). The agar was then melted in a microwave oven and filtered over a tea strainer. The remaining hyphae were rinsed with hot water and excess water was removed by placing the tea strainer on filter paper. Fungal hyphae were stored in a micro-centrifuge tube at − 80 °C until freeze drying. Hyphae were subjected to freeze drying for 24 h at − 50 ± 2 °C, and dry weight was measured.

Headspace samples were analysed by gas chromatography equipped with a thermo-desorption unit (Ultra 50:50, Markes, Llantrisant, UK) and coupled to a mass spectrometer (Thermo Fisher Scientific, Waltham, MA, USA). Fungal VOCs were desorbed from the Tenax in the thermo-desorption trap unit by heating from 25 to 250 °C (5 min hold) at a rate of 60 °C min^−1^ in splitless mode. Released compounds were focused at 0 °C in a cold trap (ID 1.80 mm) filled with Tenax and charcoal. By flash heating of the cold trap to 280 °C at 40 °C s^−1^ (hold 10 min), VOCs were transferred to the analytical column (30 m × 0.25 mm ID, 1 μm film thickness, DB-5, Phenomenex, Torrence, CA, USA) for 4 min at a constant flow of 1 ml min^−1^. The oven temperature programme started at 40 °C and immediately rose at a rate of 5 °C min^−1^ to 280 °C (hold 4 min). Column effluent was ionized by electron impact ionization at 70 eV. Mass scanning was carried out from *m*/*z* 35 to 300 at 4.70 scans s^−1^.

The detected VOCs were identified by comparison of the mass spectra with those of NIST (National Institute of Standards and Technology, USA), Wiley libraries, and the Wageningen Mass Spectral Database of Natural Products, by comparing the experimentally calculated linear retention index with the literature values and using the mVOC 2.0 database (Lemfack et al. 2017). Only VOCs detected in the samples with a peak area fourfold higher than that of control samples (VOCs from the fungal medium alone), and detected in at least 50% of the replicates of one of the fungal species were selected for further analysis. Qualitative comparisons of VOC profiles produced by pathogenic and non-pathogenic fungi were plotted in Venn diagrams. Total ion chromatograms (TIC) were used to generate values for peak area, and the VOC profiles of pathogenic and non-pathogenic fungi were analysed by multivariate analyses through a Projection to Latent Structures Discriminant Analysis (PLS-DA) (SIMCA 15 software, Umetrics AB, Umeå, Sweden). This model was evaluated using a sevenfold cross-validation test (*N *= 200; one-way ANOVA) and with *R*^2^ and *Q*^2^ estimates. Variable importance in projection (VIP) values were generated for each compound. Compounds with a VIP > 1.2 were listed as the most important for the model.

### Short-term effects of exposure to fungal volatiles on plant growth

To investigate whether a continuous exposure to fungal volatiles affects plant growth, 1-week-old *A. thaliana* seedlings were exposed in vitro to volatiles of the different fungi for 2 weeks, and their weight was compared with that of their respective control plants (i.e., exposed only to the medium incubated for 4, 7, or 10 days) (Table S1). For this, a three-compartment Petri dish was designed in which *A.* *thaliana* seedlings and the fungi were co-cultivated, but physically separated allowing volatile-mediated interactions only, as previously described (Cordovez et al. 2017). Six *A. thaliana* seedlings were cultivated in a 9 cm ø Petri dish, whereas one of the 11 fungi was cultivated in a 3 cm ø Petri dish. The Petri dishes containing the seedlings and the fungi were enclosed inside a third 14.5 cm ø Petri dish that was sealed with plastic wrap (Darco Pack B.V., The Netherlands) and incubated vertically in a climate cabinet (21 ± 1 °C; 180 µmol light m^−2^ s^−1^; 16:8 h, L:D; 70 ± 5% R.H.). Plants were harvested after 2 weeks of exposure, and root and leaf weight of volatile-exposed and control plants were dried overnight at 55 °C and weighed. Each fungal volatile exposure was replicated six times. The increase in root and leaf dry weight, as well as the change in root:leaf dry weight ratio, of volatile-exposed plants relative to the control plants was expressed in percentages, i.e., an increase in plant weight of 0% corresponds to a similar weight as control plants. These data were statistically analysed using one-sample Student’s *t* test (*H*_0_ = 0; *α *= 0.05). A two-sample Student’s *t* test was additionally performed to statistically assess the differences of plant responses (root and shoot weight) to the pathogenicity of the volatile-emitting fungi (*α *= 0.05).

### Long-term effects of exposure to fungal volatiles on plant development

To investigate whether plant responses to fungal volatiles are maintained after temporary exposure, 1-week-old plants were exposed for 1 week to fungal volatiles in vitro as described in the previous section. Exposure was disrupted after 1 week and the plants alone were transplanted to soil. Four seedlings were randomly selected from each Petri dish and transplanted to a plastic pot (ø 10 cm, *H* = 7.8 cm) filled with a sterile mixture of sand and potting soil (1:1 v/v; Horticoop potting soil ø 2 mm sieved). Plants were grown in a greenhouse compartment until harvesting (21 ± 2 °C; 300 ± 80 µmol light m^−2^ s^−1^; 16:8 h, L:D; 70 ± 5% R.H.). Date of the first flower per pot was monitored daily and plants were harvested 17 days after transplantation. To access plant growth, roots and shoots were dried overnight at 55 °C and weighed. Each fungal volatile exposure was replicated eight times. The increase of root and shoot weight, as well as the change in ratio flower:leaf dry weight, of volatile-exposed plants relative to the control plants was expressed in percentages, i.e., an increase in plant weight of 0% corresponds to a similar weight as control plants. These data were statistically analysed using one-sample Student’s *t* test (*H*_0_ = 0; *α *= 0.05). Change in flowering time relative to control plants was likewise tested for each volatile-exposed plants using one-sample Student’s *t* test (*H*_0_ = 0; *α *= 0.05). A two-sample Student’s *t* test was additionally performed to statistically assess the differences in plant responses (plant weight and flowering time) to the pathogenicity of the volatile-emitting fungi (*α *= 0.05). Correlation between change in flower dry weight and change in flowering time of exposed plants relative to control plants was tested with Pearson correlation test.

### Effects of exposure to fungal volatiles on plant resistance to a generalist insect herbivore

To test whether a temporary exposure of plants to fungal volatiles affects plant resistance to a generalist insect, 1-week-old *A.* *thaliana* seedlings were first exposed to fungal volatiles in vitro for 1 week, and then, exposure was disrupted. Four seedlings were randomly selected from each Petri dish and transplanted to pots filled with a sterile mixture of sand and potting soil. Three days following transplantation into soil, five fresh eggs of *M. brassicae* were transferred to each of the four plants per pot, and hence, 20 eggs per pot. When fewer than 50% of the larvae hatched per pot, neonate larvae were manually added to reach a minimal larval density of 10. Larval fresh weight was determined at 3 and 7 days post-hatching (dph). Larval density was reduced to 10 larvae at 3 dph to simulate natural dispersal and predation in nature (Johansen 1997). During the period in which plants were infested with the insects, plants were covered with a plastic cylinder (Duchefa, Haarlem, the Netherlands; *H* = 14 cm, upper ø 11.5 cm, lower ø 9 cm) and that was closed with a mesh and sealed with rubber bands to prevent caterpillars from escaping. Each treatment was replicated 1–3 times per batch and in 7 consecutive batches. The change in fresh weight of larvae feeding on volatile-exposed plants relative to that of larvae feeding on their respective control plants was expressed in percentages, i.e., a change in larval weight of 0% corresponds to a similar weight as in control plants, and was statistically analysed using one-sample Student’s *t* test (*H*_0_ = 0; *α *= 0.05) at each of the two time points. In addition, a two-sample Student’s *t* test was performed to statistically analyse the differences in larval fresh weight between larvae feeding on plants previously exposed to volatiles of pathogenic fungi and that of on plants exposed to volatiles of non-pathogenic fungi (*α *= 0.05).

### Collection and quantification of fungal CO_2_

To determine the emission of fungal CO_2_ and to unravel its potential role in promoting *A. thaliana* growth, we quantified CO_2_ concentration for each fungus in the absence and presence of plants. After being pre-incubated for 4, 7, or 10 days in a 3 cm ø Petri dish containing 1/5th PDA (see Sect. “Culture of fungi, plants and insects”), fungi were enclosed individually with or without plants, and 1/5th PDA was used as control. Plant exposure was performed as described above (See Sect. “Short-term effects of exposure to fungal volatiles on plant growth”). To allow sampling of the headspace with a syringe, one 14 mm ø hole was made in each lid of the large Petri dishes (14.5 cm ø), and a butyl rubber stopper was inserted (Rubber BV, Den Haag, the Netherlands). Prior to use, these lids were sterilised by rinsing with ethanol and exposing to UV light for 15 min in a flow cabinet. A volume of 250 µL was sampled manually from the headspace of the large Petri dishes after 7 and 14 days of incubation through the lid septa, and directly injected into a Trace Ultra GC gas chromatograph (Interscience, The Netherlands). The GC was equipped with a methanizer (flame ionization detector in combination with a hydrogenation reactor which converts CO_2_ into CH_4_) and at Rt-QBOND (30 m, 0.32 mm ID, cat# 19744) capillary column. Helium was used as a carrier gas with a flow of 5 ml min^−1^ and a split ratio of 1:4. The oven temperature was set at 50 °C. The data were acquired with Chromeleon 7.02 (Thermo Scientific, Germany). CO_2_ concentration was calculated for all 11 fungi and their respective controls (medium alone pre-incubated for 4, 7, or 10 days) in the presence or absence of plants. Peak areas of CO_2_ standards at 1200 ppm and 2000 ppm in synthetic air (Westfalen AG, Germany) were used for calibration. Main effects of the fungal species and sampling time on CO_2_ concentration were tested using an ANOVA. Each fungus and control was replicated 5–8 times when enclosed alone and 2–7 times when co-cultivated with *A. thaliana* plants. At the end of the 14 days of co-cultivation, all plants were harvested, dried, and weighed. Correlation between the average plant dry weight upon co-cultivation for 14 days with the fungi and the average CO_2_ concentration measured after 14 days when the fungi were enclosed alone was tested with a Pearson correlation test.

## Results

### Profiling of fungal volatile organic compounds

**Fig. 1:**
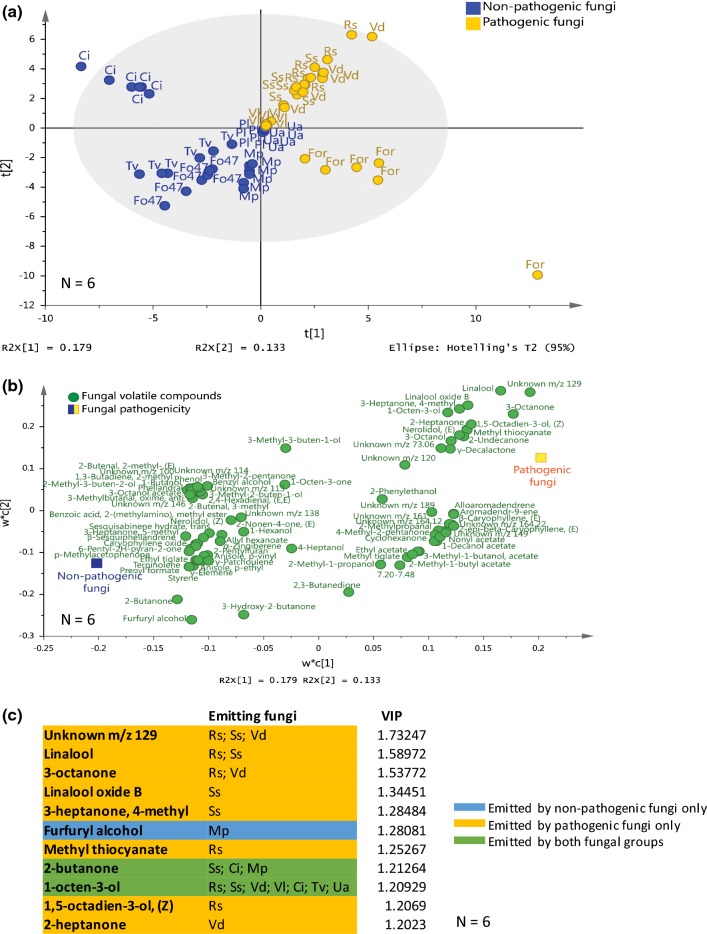
Projection to Latent Structures Discriminant Analysis (PLS-DA) of volatile organic compounds (VOCs) collected from the headspace of pathogenic and non-pathogenic fungi of *Arabidopsis thaliana*. **a** Grouping pattern of samples according to the first two principal components and the Hotelling’s T2 ellipse confining the confidence region (95%) of the score plot. **b** Contribution of individual VOCs to the first two principal components is shown in the loading plot of the PLS-DA. **c** List of VOCs with values of variable importance in projection (VIP) > 1.2. Different letters indicate the distribution of the samples of the 11 different fungi: Ci, *Chaetomium indicum;* Fo47,  *Fusarium oxysporum* 47; For, * F. oxysporum* f.sp. *raphani*; Mp,  *Mucor plumbeus;* Pl,  *Phoma leveillei;* Rs,  *Rhizoctonia solani;* Ss, * Sclerotinia sclerotiorum;* Tv,  *Trichoderma viride;* Ua,  *Ulocladium atrum;* Vd,  *Verticillium dahliae;* Vl,  *Verticillium longisporum*

Analysis of the VOC headspace of the 11 soil-borne fungi revealed a total of 82 discrete VOCs (Table S3). Approximately 15% of these compounds were detected for pathogenic as well as non-pathogenic fungi, whereas 38% and 47% of the compounds were unique to the pathogenic and non-pathogenic fungi, respectively (Fig. S2a). Among the pathogenic fungi, 58% of the compounds were unique to a fungal species, and 42% of the compounds were detected in the VOC profiles of at least two different species (Fig. S2b). There was no VOC that was common to all pathogenic fungi. Among the non-pathogenic fungi, 51% of the compounds were unique to a species, and 49% of the compounds were detected in at least two different fungi (Fig. S2c). Three co-eluted alcohols (3-methyl-1-butanol, 2-methyl-1-butanol, and 1-pentanol) were detected in the VOC profiles of all non-pathogenic species, but were also found in the VOC profiles of some pathogenic fungi (Fig. S2). Overall, the VOC profiles of the pathogenic fungi separated from the profiles of the non-pathogenic fungi: 18% and 13% of the total variance were explained by the first and second principal components, respectively (Fig. 1; PLS-DA; *R*^2^ = 0.6; *Q*^2^= 0.6, *P*_CV ANOVA_ < 0.001). Eleven VOCs had a VIP > 1.2 and these contributed the most to the separation between VOC profiles of pathogenic and non-pathogenic fungi (Fig. 1c and Table S4). The vast majority of these VOCs was detected only in pathogenic fungi. Thus, the VOC profiles of pathogenic and non-pathogenic fungi can be clearly discriminated.

### Short-term effects of continuous exposure to fungal volatiles on plant growth

**Fig. 2:**
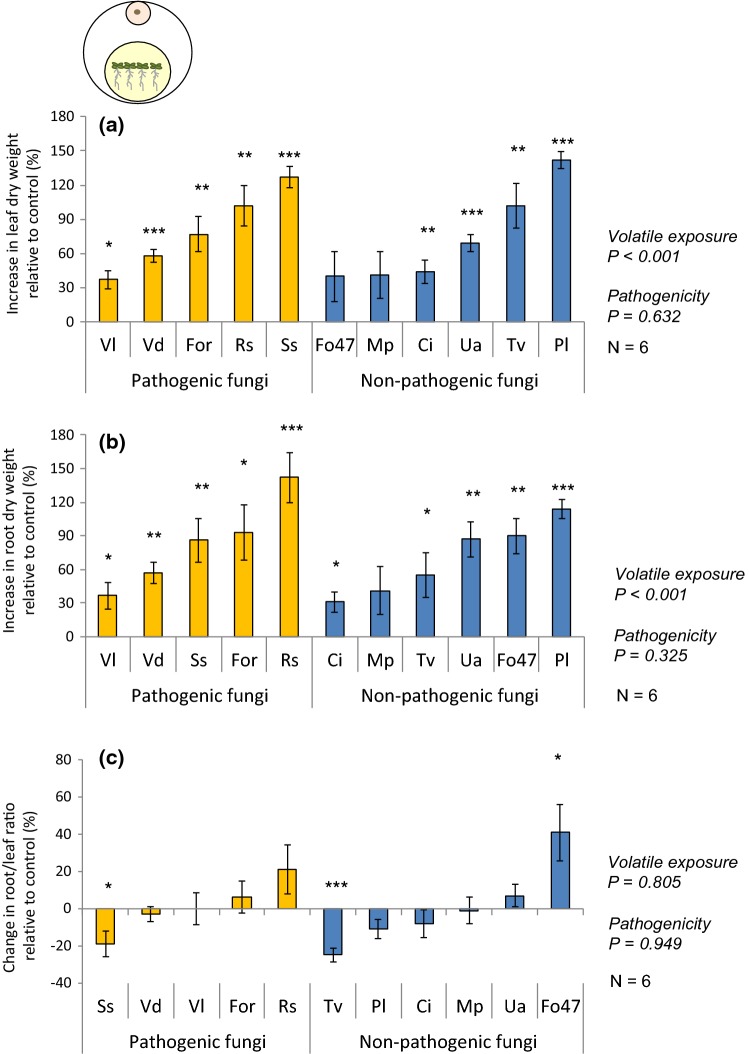
Increase in **a** leaf and **b** root dry weight (mean % ± SE), and **c** change in root:leaf ratio (mean % ± SE) of *Arabidopsis thaliana* after 2 weeks of in vitro exposure to fungal volatiles. Data are shown as relative to control plants; an increase of 0% in plant weight or ratio corresponds to a similar weight or ratio as in control plants. Ci, *Chaetomium indicum;* Fo47,  *Fusarium oxysporum* 47; For, * F. oxysporum* f.sp. *raphani;* Mp,  *Mucor plumbeus;* Pl,  *Phoma leveillei;* Rs,  *Rhizoctonia solani;* Ss, * Sclerotinia sclerotiorum;* Tv,  *Trichoderma viride;* Ua,  *Ulocladium atrum;* Vd,  *Verticillium dahliae;* Vl,  *Verticillium longisporum*. Main effect of the volatile exposure was tested using one-sample Student’s *t* test (*H*_0_ = 0), and difference of plant weight increase in response to volatiles of different fungal pathogenicity was tested using two-sample Student’s *t* test at α = 0.05. Asterisks indicate statistical differences with the respective control plants (*P < 0.05; **P < 0.01; ***P < 0.001) using one-sample Student’s *t* test (*H*_0_ = 0)

To test whether fungal volatiles affect plant growth, 1-week-old *A. thaliana* seedlings were exposed in vitro to volatiles of the different fungi for 2 weeks, and plant weight of volatile-exposed plants was compared with that of their respective control plants (Fig. 2 and Fig. S3b and c). Plant exposure to fungal volatiles had a positive main effect on both leaf and root weight (Fig. 2a, b; one-sample Student’s *t* test; *H*_0_ = 0; *P*_leaf_< 0.001; *P*_root_< 0.001), and this increase was similar for leaves and roots (Fig. 2c; one-sample Student’s *t* test; *H*_0_ = 0; *P *= 0.805). Only plants exposed to volatiles of *M. plumbeus* did not exhibit a significant increase in leaf nor root weight (Fig. 2a, b; one-sample Student’s *t* test; *H*_0_ = 0; *P*_leaf_ = 0.179; *P*_root_ = 0.194). Plants exposed to volatiles of *T.* *viride* and of *S. sclerotiorum* had a reduced root:leaf ratio relative to control plants (Fig. 2c; one-sample Student’s *t* test; *H*_0_ = 0; *P*_*T.* *viride*_ = 0.001; *P*_*S.* *sclerotiorum*_ = 0.046). In contrast, plants exposed to volatiles of *F. oxysporum* 47 had a higher root:leaf ratio relative to their respective control plants (Fig. 2c; one-sample Student’s *t* test; *H*_0_ = 0; *P *=0.041). The effects of fungal volatiles on plant growth were not associated with the pathogenicity of the fungus. Shoot and root dry weight, as well as the root:leaf ratio, did not differ between plants exposed to volatiles of pathogenic fungi and those of non-pathogenic fungi (Fig. 2a–c; two-sample Student’s *t* test; *P*_leaf_ = 0.632; *P*_root_ = 0.325; *P*_ratio_ = 0.949).

### Long-term effects of temporary exposure to fungal volatiles on plant development

**Fig. 3:**
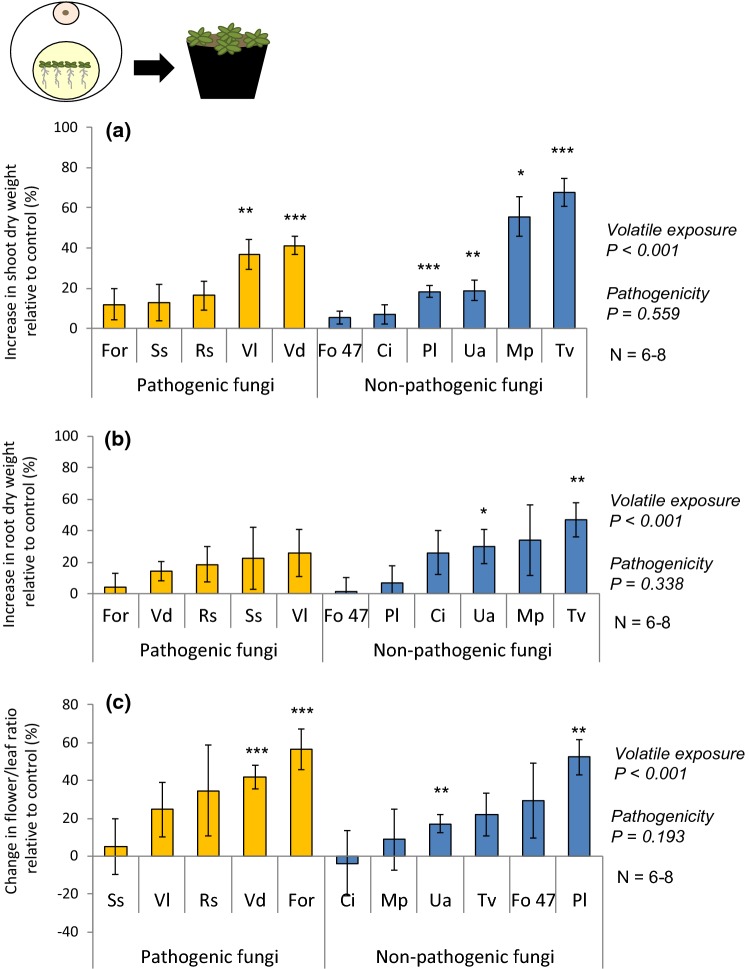
Increase in **a** shoot and **b** root dry weight (mean % ± SE), and **c** change in flower:leaf ratio (mean % ± SE) of *Arabidopsis thaliana* exposed temporary to fungal volatiles in vitro, and subsequently grown in soil for 2.5 weeks. Data are shown as relative to control plants; an increase of 0% in plant weight or ratio corresponds to the same weight or ratio as in control plants. Ci,  *Chaetomium indicum;* Fo47,  *Fusarium oxysporum* 47; For, * F. oxysporum* f.sp. *raphani;* Mp,  *Mucor plumbeus;* Pl,  *Phoma leveillei;* Rs,  *Rhizoctonia solani;* Ss *, Sclerotinia sclerotiorum;* Tv,  *Trichoderma viride;* Ua,  *Ulocladium atrum;* Vd,  *Verticillium dahliae;* Vl,  *Verticillium longisporum*. Main effect of the volatile exposure was tested using one-sample Student’s *t* test (*H*_0_ = 0), and difference of plant weight increase in response to volatiles of different fungal pathogenicity was tested using two-sample Student’s *t* test at *α* = 0.05. Asterisks indicate statistical differences with the respective control plants (*P < 0.05; **P < 0.01; ***P < 0.001) using a one-sample Student’s *t* test (*H*_0_ = 0)

**Fig. 4:**
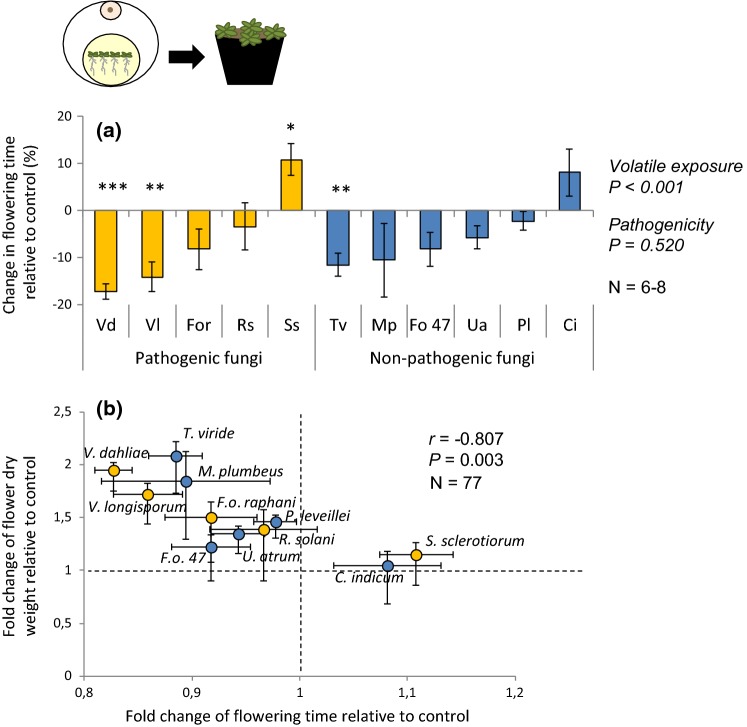
**a** Change in flowering time (mean % ± SE) of *Arabidopsis thaliana* exposed temporary to fungal volatiles in vitro, and subsequently grown in soil 2.5 weeks. **b** Pearson correlation between the fold change of flower dry weight (mean % ± SE) and flowering time (mean % ± SE) of *A.* *thaliana* exposed to the fungal volatiles relative to control. Ci,  *Chaetomium indicum;* Fo47,  *Fusarium oxysporum* 47; For, * F. oxysporum* f.sp. *raphani;* Mp,  *Mucor plumbeus;* Pl,  *Phoma leveillei;* Rs,  *Rhizoctonia solani;* Ss, * Sclerotinia sclerotiorum;* Tv,  *Trichoderma viride;* Ua,  *Ulocladium atrum;* Vd,  *Verticillium dahliae;* Vl,  *Verticillium longisporum.* Main effect of the volatile exposure was tested using a one-sample Student’s *t* test (*H*_0_ = 0), and difference of flowering time in response to volatiles of different fungal pathogenicity was tested using two-sample Student’s *t* test at *α* = 0.05. Asterisks indicate statistical differences with the respective control plants (*P < 0.05; **P < 0.01; ***P < 0.001) using one-sample Student’s *t* test (*H*_0_ = 0). For the Pearson correlation, dash lines represent the control plants

To test whether plant responses to fungal volatiles can be sustained after temporary exposure, we assessed the weight of plants exposed in vitro to fungal volatiles for only 1 week after which they were transplanted to soil in the absence of the volatile-emitting fungus (Fig. 3 and Fig. S3b and c). Plant exposure to fungal volatiles had a positive main effect on shoot (Fig. 3a; one-sample Student’s *t* test; *H*_0_ = 0; *P *<0.001) and root dry weight (Fig. 3b; one-sample Student’s *t* test; *H*_0_ = 0; *P *<0.001). Particularly, plants exposed to volatiles of *T. viride* and *U. atrum* had an increase in both shoot (Fig. 3a; one-sample Student’s *t* test; *H*_0_ = 0; *P*_*T. viride*_ < 0.001; *P*_*U. atrum*_ = 0.010) and root dry weight (Fig. 3b; one-sample Student’s *t* test; *H*_0_ = 0; *P*_*T. viride*_ = 0.004; *P*_*U. atrum*_ = 0.031). In addition, plant exposure to fungal volatiles had a positive main effect on the flower:leaf ratio (Fig. 3c; one-sample Student’s *t* test; *H*_0_ = 0; *P * < 0.001). Shoot and root dry weight as well as the flower:leaf ratio did not differ between plants exposed to volatiles of pathogenic fungi and that of non-pathogenic fungi (Fig. 3a–c; two-sample Student’s *t* test; *P*_shoots_ = 0.559; *P*_roots_ = 0.338; *P*_ratio_ = 0.193). Overall, *A. thaliana* plants exposed to fungal volatiles produced flowers sooner than control plants (Fig. 4a; one-sample Student’s *t* test; *H*_0_ = 0; *P *<0.001). Only volatiles of *S. sclerotiorum* delayed flowering (Fig. 4a; one-sample Student’s *t* test; *H*_0_ = 0; *P * = 0.019). No association with pathogenicity was found (Fig. 4a; two-sample Student’s *t* test; *P * = 0.520). Flowering time negatively correlated with flower dry weight (Fig. 4b; Pearson correlation; *N* = 77; *r* = − 0.807; *P* = 0.003).

### Effects of temporary exposure to fungal volatiles on plant resistance to a generalist insect herbivore

**Fig. 5:**
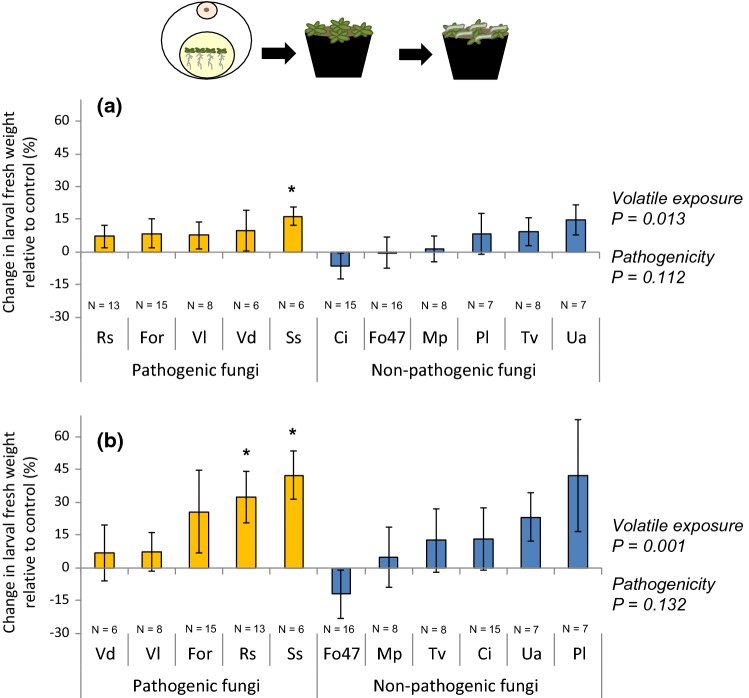
Change in larval fresh weight (mean ± SE) of *Mamestra brassicae* at **a** 3 days post-hatching and **b** 7 days post-hatching when feeding on *Arabidopsis thaliana* exposed temporary to fungal volatiles in vitro, and subsequently grown in soil. Ci,  *Chaetomium indicum*; Fo47,  *Fusarium oxysporum* 47; For, *F. oxysporum* f.sp. *raphani;* Mp,  *Mucor plumbeus*; Pl,  *Phoma leveillei;* Rs,  *Rhizoctonia solani;* Ss, * Sclerotinia sclerotiorum;* Tv,  *Trichoderma viride;* Ua,  *Ulocladium atrum;* Vd,  *Verticillium dahliae;* Vl,  *Verticillium longisporum.* Main effect of the volatile exposure was tested using a one-sample Student’s *t* test (*H*_0_ = 0), and difference of larval fresh weight between plants exposed to volatiles of different fungal pathogenicity was tested using two-sample Student’s *t* test at *α* = 0.05. Asterisks indicate statistical differences with the respective control plants (*P < 0.05; **P < 0.01; ***P < 0.001) using one-sample Student’s *t* test (*H*_0_ = 0). *N* indicates the number of pots that were infested with 20 larvae

To test whether a temporary exposure of plants to fungal volatiles affects plant resistance to a generalist insect, we assessed the weight of larvae feeding on plants previously exposed to fungal volatiles and on control plants at two time points (Fig. 5 and Fig. S3b and d). Plant exposure to fungal volatiles had a positive main effect on larval weight at 3 days post-hatching (dph) (Fig. 5a; one-sample Student’s *t* test *H*_0_ = 0; *P *= 0.013) and at 7 dph (Fig. 5b; one-sample Student’s *t* test *H*_0_ = 0; *P *= 0.001). In particular, larvae feeding on plants previously exposed to volatiles of *S. sclerotiorum* were larger at 3 dph than larvae fed on control plants (Fig. 5a; one-sample Student’s *t* test *H*_0_ = 0; *P*_*S.* *sclerotiorum*_ = 0.011). In addition, larvae feeding on plants previously exposed to volatiles of *S. sclerotiorum* and *R. solani* were also larger than those feeding on control plants at 7 dph (Fig. 5b; one-sample Student’s *t* test *H*_0_ = 0; *P*_*S. sclerotiorum*_ = 0.013; *P*_*R. solani*_ = 0.018). Larval fresh weight, at 3 dph and 7 dph, did not differ between larvae feeding on plants exposed to volatiles of pathogenic fungi and that of plants exposed to volatiles of non-pathogenic fungi (Fig. 5a and b; two-sample Student’s *t* test; *P*_3dph_ = 0.112; *P*_7dph_ = 0.132). Larval weight at 7 dph was correlated with shoot dry weight of infested plants previously exposed to fungal volatiles (Fig. S4a; Pearson correlation; *N* = 68; *r * = 0.330; *P * = 0.006) but not with the difference of shoot weight between uninfested and infested plants (Fig. S4b; Pearson correlation; *N* = 62; *r * = 0.045; *P * = 0.726).

### Collection and quantification of fungal CO_2_

**Fig. 6:**
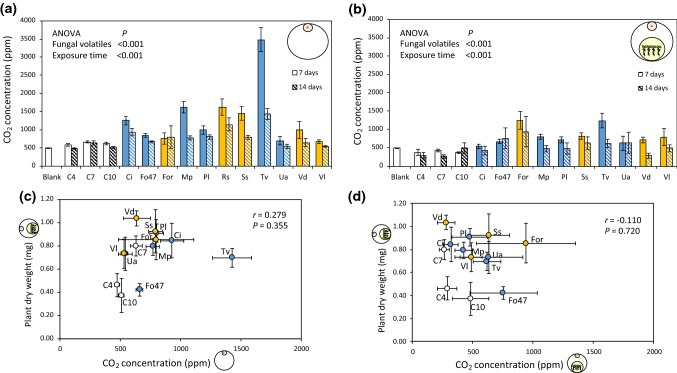
CO_2_ concentration (mean ± SE) measured for the 11 fungi and their respective controls when **a** enclosed alone, and **b** co-cultivated with *Arabidopsis thaliana* seedlings for 7 and 14 days. Pearson correlation between the average plant dry weight (mean ± SE) upon co-cultivation for 14 days and **c** the average CO_2_ concentration (mean ± SE) measured after 14 days when the fungi were enclosed alone, and **d** the average CO_2_ concentration (mean ± SE) measured after 14 days when the fungi were co-cultivated with plants. Blank, empty Petri dish; C4, C7 and C10, medium alone pre-incubated for 4, 7, and 10 days; Ci,  *Chaetomium indicum;* Fo47,  *Fusarium oxysporum* 47; For, * F. oxysporum* f.sp. *raphani;* Mp,  *Mucor plumbeus;* Pl,  *Phoma leveillei;* Rs,  *Rhizoctonia solani;* Ss, * Sclerotinia sclerotiorum;* Tv,  *Trichoderma viride;* Ua,  *Ulocladium atrum;* Vd,  *Verticillium dahliae;* Vl,  *Verticillium longisporum*. Upon co-cultivation with plants, each fungal volatile exposure was replicated 2–7 times, and when the fungus was incubated alone, each volatile exposure was replicated 5–8 times. Due to fungal overgrowth on plant compartment, exposure with *R. solani* volatiles was excluded from the analysis upon co-cultivation with *A. thaliana.* Main effects of the fungal volatiles and exposure time were tested using ANOVA. Detailed output of the pairwise differences between the fungal volatile exposures is reported in the electronic supplemental material (Fig. S5)

To assess the potential contribution of fungal CO_2_ to the plant growth-promoting effects observed in vitro, we quantified the CO_2_ concentration for each fungus and control (i.e., medium alone) in the absence and presence of *A. thaliana* plants for 7 and 14 days (Fig. 6). CO_2_ concentration differed between fungi when they were growing alone (Fig. 6a; ANOVA; *P *< 0.001) as well as in co-cultivation with *A. thaliana* (Fig. 6b; ANOVA; *P *< 0.001). Detailed output of the pairwise differences between the fungal volatile exposures is reported in the electronic supplemental material (Fig. S5). In both situations, i.e., when fungi were enclosed alone or co-cultivated with plants, CO_2_ concentration overall decreased with time (Fig. 6a, b; ANOVA; *P *< 0.001). Plant dry weight after 14 days of co-cultivation with fungi did not correlate with CO_2_ concentration measured after 14 days when the fungi were enclosed alone (Fig. 6c; Pearson correlation test; *r *= 0.279; *P *= 0.355) nor with CO_2_ concentration measured after 14 days of co-cultivation (Fig. 6d; Pearson correlation test; *r *= − 0.110; *P *= 0.720).

## Discussion

We studied the effects of volatiles emitted by pathogenic and non-pathogenic fungi on plant development and resistance to insects, and found that, overall, fungal volatiles increased plant weight and accelerated plant development, irrespective of the pathogenicity of the fungus and of the fungal CO_2_ emission. Based on these phenotypic changes, plants do not seem to discriminate between volatiles of pathogenic and non-pathogenic soil-borne fungi, despite distinct compositional differences in the VOC profiles of these two groups. Interestingly, volatiles of pathogenic and non-pathogenic fungi can both affect plant resistance and development. Overall, *A. thaliana* plants exposed to fungal volatiles flowered earlier, and plants exposed to volatiles of some fungi in particular became more susceptible to herbivory by generalist caterpillars. These results indicate that plant exposure to fungal volatiles can affect plant development and resistance.

The profiling of fungal VOCs showed that some compounds were species-specific, whereas a few compounds were present in the VOC profiles of both pathogenic and non-pathogenic fungi, such as the typical mushroom odour, 1-octen-3-ol. Previous studies showed that soil-borne fungi and bacteria emit unique VOCs, which can be used as microbial signatures (De Lucca et al. 2010; Bos et al. 2013; Müller et al. 2013; Cordovez et al. 2015). More specifically, fungal VOCs may allow the classification of different fungal lifestyles, including pathogenicity (Müller et al. 2013). It is important to bear in mind, however, that the denomination of “pathogen” is specific to a plant host-fungus system and may differ in another system. In the present study, we did not detect VOCs that were common to all pathogenic fungi and that were absent in the VOC profiles of non-pathogenic fungi. Similarly, we did not detect VOCs that were common to all non-pathogenic fungi and that were absent in the VOC profiles of pathogenic fungi tested in this study. Together, these data indicate that a large diversity of VOCs is emitted by different soil-borne fungal species. Differences between the VOC profiles of pathogenic and non-pathogenic fungi were mostly explained by compounds exclusively emitted by the pathogenic fungi we tested. These findings highlight the importance of the presence/absence of these VOCs in discriminating the profiles of the pathogenic and non-pathogenic fungi in this system. The distinct VOC profiles observed in the conditions tested provide a solid empirical basis to further investigate if plants respond differently upon exposure to volatiles from pathogenic and non-pathogenic fungi.

Interactions among plants and fungi are among the oldest interactions on Earth, and microbial volatiles act as an ancient cue involved in plant–microbe interactions, which was shaped during evolutionary history and established before the development of higher plants (Sharifi and Ryu 2018). Therefore, it is plausible to expect plants to have evolved differential responses to volatile cues from pathogenic and non-pathogenic microorganisms surrounding them, in particular when volatile profiles are distinct, as plants could benefit from anticipating the arrival of pathogens and mutualists. Interestingly, although only pathogenic fungi represent an actual threat to plant fitness, plants did not differentially respond to fungal volatiles in the present study. Fungal VOCs were collected for 2 h in the absence of plants, and therefore, this snapshot does not include possible changes in fungal VOC emission in the presence of a plant. Potentially, fungi in the vicinity of plants may perceive the presence of their hosts (Hegedus and Rimmer 2005; Chagas et al. 2018), e.g., via root volatiles, and respond by changing their own VOC emission (Venturi and Keel 2016). A change in fungal VOCs, e.g., emission of similar VOCs found in non-pathogenic microorganisms or in roots (Schenkel et al. 2015), may render pathogens undetectable to plants that, consequently, cannot distinguish pathogenic from non-pathogenic microorganisms. This VOC “dialogue” between fungi and plants may be dynamic over time (Fincheira and Quiroz 2018). Further real-time analysis of the fungal VOCs over a longer period of time in the absence and presence of plants may shed light on this two-way interaction (van Dam et al. 2012).

In the present study, plant weight was overall enhanced and flowering was accelerated upon exposure to fungal volatiles, irrespective of the pathogenicity of the fungus and its CO_2_ emission. These results indicate that plants respond to a wide range of volatiles produced by different fungal species, and are in line with the results of recent studies that have shown a critical role of microbial volatiles in plant growth and health (Bitas et al. 2013; Kanchiswamy et al. 2015; Piechulla et al. 2017; Sharifi and Ryu 2018). In addition to fungal VOCs, fungal CO_2_ may also accumulate in closed systems and lead to plant growth promotion (Kai and Piechulla 2009; Piechulla and Schnitzler 2016). Our data show that enclosure of fungi alone, indeed, increased the CO_2_ concentration in our experimental set-up, and that CO_2_ emission differs between the fungi. However, CO_2_ concentration overall decreased with time in the presence but also in the absence of plants. We speculate that this is most likely due to the fact that the plastic used for wrapping the Petri dishes was not fully airtight and allowed some CO_2_ diffusion. Hence, the set-up used for the volatile exposure is in fact not completely closed. In the present study, plant dry weight did not correlate with CO_2_ concentrations measured when the fungi were enclosed in the presence nor in the absence of plants. In fact, plants exposed to the highest CO_2_-emitting fungi did not show the strongest growth promotion. These results indicate that increased CO_2_ in our experimental set-up was not the main driver for the observed plant growth-promoting effects. Additional evidence shows that, in closed system, fungal CO_2_ alone does not trigger growth promotion to the same extent as when plants are also exposed to VOCs (Sánchez-López et al. 2016; Cordovez et al. 2017). However, we cannot exclude that other inorganic volatile compounds may have contributed as well to the plant responses observed in our study (Sánchez-López et al. 2016; García-Gómez et al. 2018).

Our study presents evidences that plants can have long-term effects following volatile exposure, which affects plant developmental processes. A temporary plant exposure to the fungal volatiles was sufficient to observe enhanced plant growth and accelerated development. To date, only a few studies have correlated an increased plant weight with a change in developmental traits (Xie et al. 2009; Hung et al. 2013; Sánchez-López et al. 2016). Interestingly, the greater the increase in flower weight, the earlier plants flowered, which implies faster development rather than merely growth promotion. Acceleration of flowering is a common plant response to stress among short-lived plants, and therefore, our findings suggest that plants may have perceived fungal volatiles as a ‘warning’. Diverse biotic stresses have been shown to trigger faster flowering (Lucas-Barbosa et al. 2013; Pashalidou et al. 2013), and exposure of plants to microbial volatiles at the seedling stage may likewise affect the transition from vegetative to reproductive stages (Sánchez-López et al. 2016). This reproductive escape strategy might be particularly important for annual plant species to prevent pathogens to colonise and infect the plant (Douglas 2008). Early flowering might be costly for plants that rely on insect pollination, because it may desynchronise the presence of pollinators and the flowering state of the plant (Kudo and Ida 2013; Petanidou et al. 2014). However, *A. thaliana* that we studied does not rely on pollinators for reproduction and, therefore, acceleration of development in response to a potential threat seems to be an advantageous strategy for this self-compatible short-lived species to ensure reproduction.

In the present study, direct resistance of *A. thaliana* seedlings against herbivory by *M. brassicae* was overall negatively affected by the volatile exposure, and led to a significantly better *M. brassicae* performance on plants exposed to volatiles of some fungi than on control plants. Increased plant weight could have provided the herbivore with more plant material that, in turn, can enhance its performance (Aziz et al. 2016). In this study, larval weight was positively correlated with the shoot fresh weight of infested plants but not with the difference of shoot weight between uninfested and infested plants, suggesting that the larvae did not grow faster by consuming more plant material. Instead, these results show a change in the plant primary and secondary metabolites that leads to increased plant susceptibility. Indeed, upon perception of microbial volatiles, plant chemistry can be altered, in particular secondary metabolites involved in resistance such as glucosinolates, rendering plants more or less resistant to a subsequent attacker (Aziz et al. 2016). Alternatively, *A. thaliana* plants exposed to the volatiles emitted by *R. solani* and *S. sclerotiorum* could have also become more nutritious for the caterpillars, e.g., due to an alteration of the leaf carbon:nitrogen ratio or starch accumulation (Ezquer et al. 2010), which, in turn, can enhance the conversion of plant material into larval body mass (Lincoln et al. 1993). We hypothesize that these plant responses may result from the perception of linalool, i.e., the only VOC that was exclusively detected in the VOC profiles of *R. solani* and *S. sclerotiorum.* This monoterpene alcohol can positively and negatively affect insect attraction to plants (Aharoni et al. 2003; Xiao et al. 2012), but the effects on plant direct resistance against herbivorous insects remain to be elucidated (Ton et al. 2006; Xiao et al. 2012). Interestingly, linalool may also negatively affect *A. thaliana* growth (Aharoni et al. 2003). Further metabolomic analyses of plants exposed to volatiles of *R. solani* and *S. sclerotiorum* and to linalool alone will shed light on the mechanisms underlying such increase of *M. brassicae* performance on these plants.

In conclusion, our results show that plants do not respond differentially to volatile cues from the tested pathogenic compared to non-pathogenic fungi, although these fungal groups emitted distinct VOC profiles. Plants respond with accelerating development, sometimes at the cost of reduced resistance to insect herbivores. Despite the reduced resistance in some volatile-exposed plants, plants can potentially and ultimately benefit from accelerated development by sustaining fitness and by escaping from potential threats. Similarly to the initial stages of microbe-associated molecular pattern recognition, plants may not be able to discriminate pathogenic fungi from non-pathogenic fungi upon perception of volatiles (Wenke and Piechulla 2013). Our findings provide new fundamental insight into plant–microbe interactions showing that despite distinct VOC profiles, volatiles of pathogenic and non-pathogenic fungi can both affect plant resistance and development. These findings have significant implications for the understanding of how plants respond to chemical cues from soil-borne fungi in terms of growth and resistance, and how these responses may be exploited to improve durable production of crops.

## Electronic supplementary material

Below is the link to the electronic supplementary material.

Supplementary material 1 (PDF 1201 kb)
