# Supplementary material for: Volatiles of pathogenic and non-pathogenic soil-borne fungi affect plant development and resistance to insects
Source: Oecologia. 2019 Jun 15;190(3):589–604. doi: 10.1007/s00442-019-04433-w (PMC6647456; doi:10.1007/s00442-019-04433-w)
Supplement: Supplementary file 1 — Supplementary material 1 (PDF 1201 kb) [file 442_2019_4433_MOESM1_ESM.pdf]

Oecologia **Electronic Supplemental Material**

Volatiles of pathogenic and non-pathogenic soil-borne fungi affect plant development and resistance to insects

Kay Moisan*[1, 2], Viviane Cordovez[2], Els M. van de Zande[1], Jos M. Raaijmakers[2, 3], Marcel Dicke[1], Dani Lucas-Barbosa[1,4]

[1]Laboratory of Entomology, Wageningen University and Research, Wageningen, the Netherlands;

[2]Department of Microbial Ecology, Netherlands Institute of Ecology, Wageningen, the Netherlands;

[3]Institute of Biology, Leiden University, Leiden, the Netherlands

[4]Current address: Bio-communication and Ecology, ETH Zürich, Zürich, Switzerland

*Correspondence: kay.moisan@wur.nl, +31 317 484148

The following Electronic Supplemental Material is available for this article:

Supplemental Materials & Methods and Results: Pathogenicity assay

Fig S1 Disease incidence of *Arabidopsis thaliana* inoculated with the soil-borne fungal pathogens *Rhizoctonia solani, Sclerotinia sclerotiorum* and *Verticillium longisporum*

Fig S2 Venn diagrams comparing the qualitative difference of the headspace volatile organic compounds detected for pathogenic and non-pathogenic fungi

Fig S3 Detailed output of the statistical tests performed in this study

Fig S4 Correlation between *Mamestra brassicae* larval weight and shoot weight of infested plants previously exposed to fungal volatiles, and with the difference of shoot weight between uninfested plants and infested plants previously exposed to fungal volatiles.

Fig S5 Statistical output of the pairwise comparisons between the fungal volatile exposures in the absence and presence of *Arabidopsis thaliana* seedlings, using Tukey post-hoc tests.

Table S1 Fungi and information on accession number, abbreviations used in this study, source, pathogenicity and incubation time

Table S2 List of fungal pathogens used in this study and their corresponding host range specificity, disease symptoms and infection mechanisms

Table S3 Volatile organic compounds quantified in the headspace of the pathogenic fungi and non-pathogenic fungi using total ion chromatograms

Table S4 List of the variable importance in projection (VIP) values for each volatile organic compound of the fungal headspace

Pathogenicity assay

Materials & Methods

Pathogenicity of specific strains used here (*R. solani, S. sclerotioum* and *V. longisporum*) to *A. thaliana* Col-0 which had not been previously described in the literature was tested (Table S1). To test this, the fungi were inoculated on 17 day-old *A. thaliana*, and plants were monitored for infection 1 and 2 weeks after inoculation. All plants were grown individually in plastic pots (7 x 7 x 8 cm) filled with 100% sterile sand supplemented with half-strength Hoagland solution (Hoagland and Arnon, 1950), and kept in a climate chamber (24 ± 1 °C; 180 µmol light m^{-2} s^{-1}; 16:8 h, L:D; 70 ± 5% R.H.) until plants had developed six fully expanded leaves. For *R. solani*, a mycelium plug (5 mm) was buried in the sand directly in contact with the roots. For *S. sclerotiorum*, a mycelium plug was inoculated on the 5th leaf of the *A. thaliana* rosette. For *V. longisporum*, plants were up-rooted, roots were incubated in a conidial suspension (10^6 conidia ml^{-1}) for 3 min and subsequently replanted. Control plants were inoculated either with a plug of agar only or by dipping roots into water. After the fungal inoculation, plants remained covered with a plastic lid for one week to ensure high humidity, favourable for the fungal infection. Each inoculation was replicated six times. Disease incidence was calculated as the percentage of diseased leaves over the total number of leaves when regarding *R. solani* and *S. sclerotiorum*, whereas for *V. longisporum*, shoot dry weight was measured. Fungus-inoculated plants and control plants (non-inoculated) were compared using the two-sample Student's *t*-test (α = 0.05).

Results

Rhizoctonia-inoculated plants displayed yellow/white leaves already at 7 days after inoculation (dai), but symptoms became stronger at 14 dai (Fig. S1a; two-sample Student's *t*-test; P = 0.016). Symptoms of *Sclerotinia*-inoculated *A. thaliana* were relatively more subtle, and were visible only at the spot of inoculation on the leaf at 8 dai (Fig. S1b; two-sample Student's *t*-test; P = 0.024). Plants inoculated with *V. longisporum* were smaller than control plants at 15 dai (Fig. S1c; two-sample Student's *t*-test; P = 0.003).

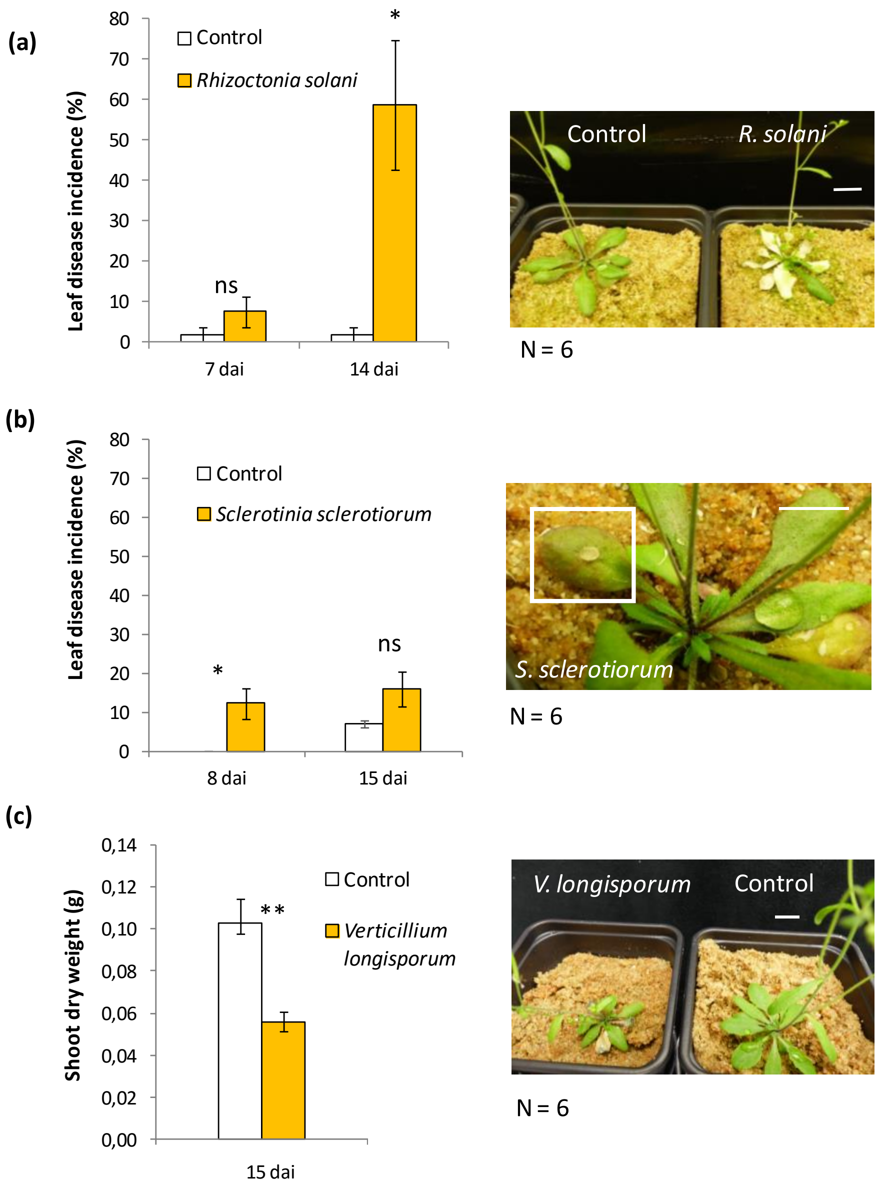

Fig S1 Disease incidence of *Arabidopsis thaliana* inoculated with the soil-borne fungal pathogens *Rhizoctonia solani Sclerotinia sclerotiorum* and *Verticillium longisporum*. Disease incidence was calculated as the percentage of leaves (± SE) with disease symptoms at 7 and 14 days after inoculation (dai) for plants inoculated with *R. solani* **(a),** and after 8 and 15 dai for plants inoculated with *S. sclerotiorum* **(b)**, whereas disease incidence in plants inoculated with *V. longisporum* **(c)** was determined by the shoot dry weight (± SE) at 15 dai. Asterisks indicate statistical differences (*: $P < 0.05$; **: $P < 0.01$) using two-sample Student *t*-test.

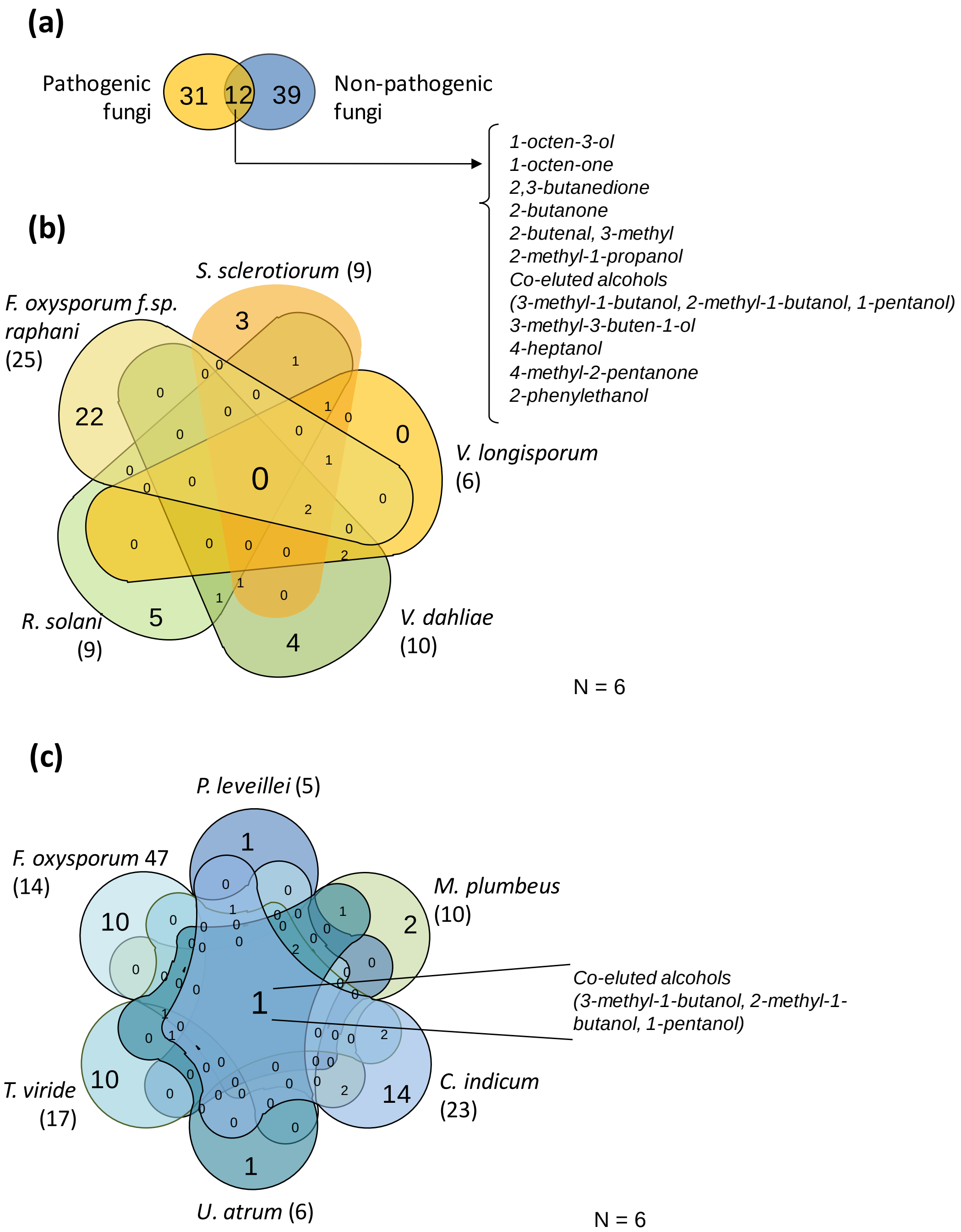

Fig S2 Venn diagrams comparing the qualitative difference of the headspace volatile organic compounds detected for pathogenic and non-pathogenic fungi. (a) Total number of unique and shared volatiles detected for pathogenic and non-pathogenic fungi, **(b)** within the pathogenic fungi, and **(c)** within the non-pathogenic fungi

(b)

			t	df	*P*-values
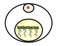	Leaf	Volatile exposure	13.285	61	< 0.001
		Pathogenicity	-0.481	60	0.632
	Root	Volatile exposure	12.331	61	< 0.001
		Pathogenicity	-0.993	60	0.325
	Root: leaf	Volatile exposure	0.248	61	0.805
		Pathogenicity	-0.064	60	0.949
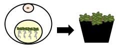	Shoot	Volatile exposure	8.915	76	< 0.001
		Pathogenicity	0.587	75	0.559
	Root	Volatile exposure	5.341	76	< 0.001
		Pathogenicity	0.965	75	0.338
	Flower: leaf	Volatile exposure	5.894	76	< 0.001
		Pathogenicity	-1.313	75	0.193
	Flowering time	Volatile exposure	-4.109	76	< 0.001
		Pathogenicity	0.646	75	0.520
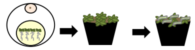	3 dph	Volatile exposure	2.516	107	0.013
		Pathogenicity	-1.603	106	0.112
	7 dph	Volatile exposure	3.381	107	0.001
		Pathogenicity	-1.518	106	0.132

(c)

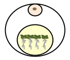

		t	df	*P*-values
Leaf	*Ci*	4.394	5	0.007
	Fo47	1.808	5	0.130
	For	5.071	5	0.004
	Mp	2.035	2	0.179
	Pl	18.462	5	<0.001
	Rs	5.728	5	0.002
	Ss	14.001	5	<0.001
	Tv	5.296	5	0.003
	Ua	9.222	5	<0.001
	Vd	10.508	5	<0.001
	Vl	4.458	4	0.011
Root	*Ci*	3.268	5	0.022
	Fo47	5.719	5	0.002
	For	3.792	5	0.013
	Mp	1.928	2	0.194
	Pl	13.830	5	<0.001
	Rs	6.415	5	0.001
	Ss	4.381	5	0.007
	Tv	2.734	5	0.041
	Ua	5.547	5	0.003
	Vd	6.103	5	0.002
	Vl	3.141	4	0.035
Root: leaf	*Ci*	-1.067	5	0.335
	Fo47	2.728	5	0.041
	For	0.714	5	0.507
	Mp	-0.200	2	0.860
	Pl	-2.251	5	0.074
	Rs	1.584	5	0.174
	Ss	-2.643	5	0.046
	Tv	-6.462	5	0.001
	Ua	1.199	5	0.284
	Vd	-0.657	5	0.540
	Vl	-0.051	4	0.962

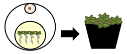

		t	df	*P*-values
Shoot	*Ci*	1.548	6	0.173
	Fo47	1.662	7	0.141
	For	1.559	7	0.163
	Mp	5.664	3	0.011
	Pl	6.333	5	0.001
	Rs	2.265	6	0.064
	Ss	1.465	6	0.193
	Tv	9.831	7	<0.001
	Ua	3.678	6	0.010
	Vd	9.115	7	<0.001
	Vl	4.887	6	0.003
Root	*Ci*	1.878	6	0.109
	Fo47	0.125	7	0.904
	For	0.448	7	0.667
	Mp	1.526	3	0.225
	Pl	0.673	5	0.531
	Rs	1.649	6	0.150
	Ss	1.152	6	0.293
	Tv	4.251	7	0.004
	Ua	2.795	6	0.031
	Vd	2.273	7	0.057
	Vl	1.701	6	0.140
Flower: leaf	*Ci*	-0.228	6	0.827
	Fo47	1.488	7	0.180
	For	5.179	7	0.001
	Mp	0.544	3	0.624
	Pl	5.674	5	0.002
	Rs	1.454	6	0.196
	Ss	0.347	6	0.740
	Tv	1.956	7	0.091
	Ua	3.740	6	0.010
	Vd	6.679	7	<0.001
	Vl	1.686	6	0.143
Flowering time	*Ci*	1.623	6	0.156
	Fo47	-2.269	7	0.058
	For	-1.940	7	0.930
	Mp	-1.351	3	0.269
	Pl	-1.129	5	0.310
	Rs	-0.678	6	0.523
	Ss	3.179	6	0.019
	Tv	-4.686	7	0.002
	Ua	-2.342	6	0.058
	Vd	-10.009	7	<0.001
	Vl	-4.416	6	0.004

(d)

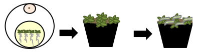

		t	df	*P*-values
3 dph	*Ci*	-1.109	14	0.286
	Fo47	-0.035	15	0.973
	For	1.269	14	0.225
	Mp	0.258	7	0.804
	Pl	0.867	6	0.419
	Rs	1.398	12	0.187
	Ss	3.926	5	0.011
	Tv	1.4	7	0.204
	Ua	2.339	6	0.058
	Vd	1.07	5	0.334
	Vl	1.185	6	0.281
7 dph	*Ci*	0.932	14	0.367
	Fo47	-1.106	15	0.286
	For	1.352	14	0.198
	Mp	0.358	7	0.731
	Pl	1.635	6	0.153
	Rs	2.732	12	0.018
	Ss	3.772	5	0.013
	Tv	0.868	7	0.414
	Ua	2.122	6	0.078
	Vd	0.541	5	0.612
	Vl	0.764	6	0.474

(a)

		t	df	*P*-values
Rhizoctonia solani	7 dai	-1.398	10	0.203
	14 dai	-3.531	10	0.016
Sclerotinia sclerotiorum	8 dai	3.216	7	0.024
	15 dai	1.359	7	0.216
Verticillium longisporum	15 dai	-4.534	7	0.003

Fig S3 Output of the Student two-samples *t*-tests and Student one-sample *t*-tests ($H_0 = 0$) performed (a) for the disease incidence, (b) for the effects of volatile exposure and pathogenicity on plant dry weight, flowering time and larvae fresh weight, (c) for the individual effects of the fungal species on plant dry weight and flowering time, and (d) for the individual effects of the fungal species on larvae fresh weight

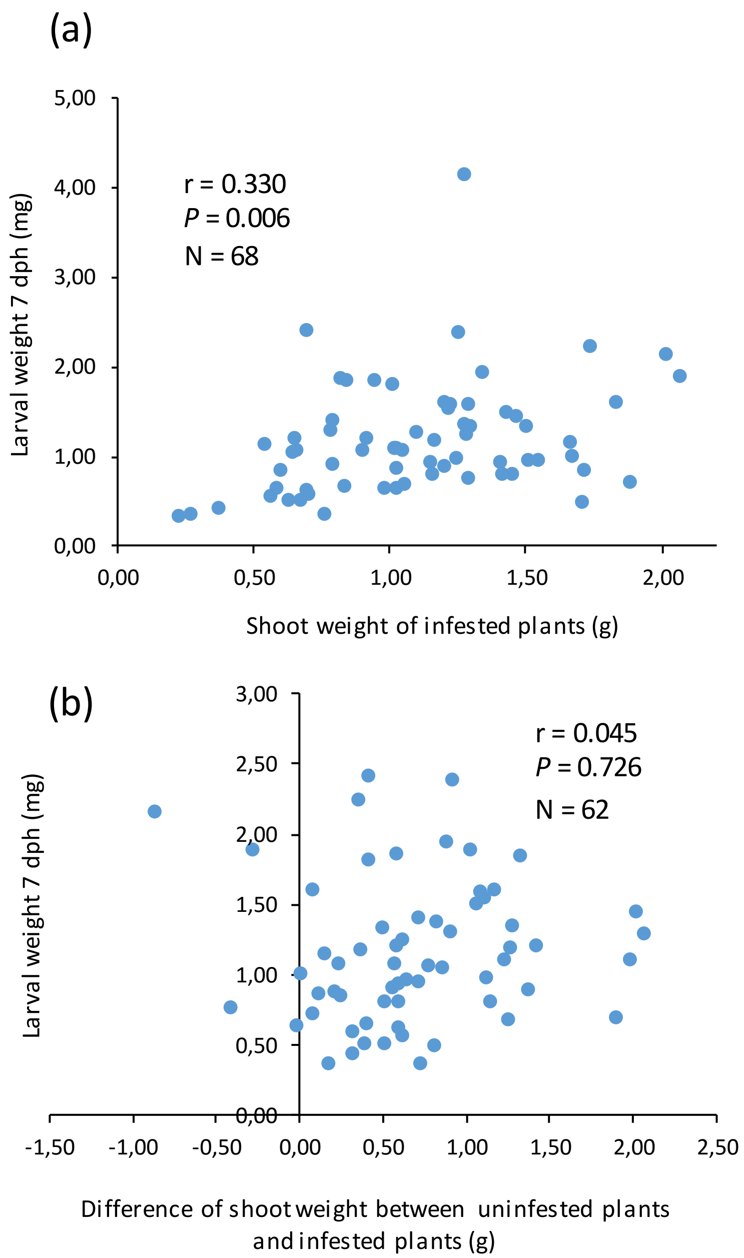

Fig S4 Pearson correlation between fresh weight of *Mamestra brassicae* larvae at 7 days post hatching and (a) shoot fresh weight of infested *Arabidopsis thaliana* previously exposed to volatiles of *Rhizoctonia solani, Chaetomium indicum, Fusarium oxysporum f.sp. raphani, Fusarium oxysporum* 47 and non-exposed plants, and (b) with the difference of shoot weight between non-infested and infested *A. thaliana* previously exposed to volatiles of *R. solani, C. indicum, F. oxysporum f.sp. raphani, F. oxysporum* 47 and non-exposed plants.

(a) 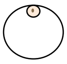

contrast	p.value	contrast	p.value	contrast	p.value	contrast	p.value
C10 - C4	1.000	C7 - Ci	0.389	Fo47 - Ss	0.727	Pl - Vl	0.925
C10 - C7	1.000	C7 - Fo47	1.000	Fo47 - Tv	<.0001	Rs - Ss	0.973
C10 - Ci	0.199	C7 - For	1.000	Fo47 - Ua	1.000	Rs - Tv	<.0001
C10 - Fo47	0.997	C7 - Mp	0.063	Fo47 - Vd	1.000	Rs - Ua	0.005
C10 - For	0.989	C7 - Pl	0.958	Fo47 - Vl	1.000	Rs - Vd	0.137
C10 - Mp	0.026	C7 - Rs	0.005	For - Mp	0.414	Rs - Vl	0.003
C10 - Pl	0.793	C7 - Ss	0.235	For - Pl	1.000	Ss - Tv	<.0001
C10 - Rs	0.002	C7 - Tv	<.0001	For - Rs	0.056	Ss - Ua	0.228
C10 - Ss	0.109	C7 - Ua	1.000	For - Ss	0.773	Ss - Vd	0.916
C10 - Tv	<.0001	C7 - Vd	0.999	For - Tv	<.0001	Ss - Vl	0.189
C10 - Ua	1.000	C7 - Vl	1.000	For - Ua	1.000	Tv - Ua	<.0001
C10 - Vd	0.972	Ci - Fo47	0.858	For - Vd	1.000	Tv - Vd	<.0001
C10 - Vl	1.000	Ci - For	0.894	For - Vl	0.999	Tv - Vl	<.0001
C4 - C7	1.000	Ci - Mp	1.000	Mp - Pl	0.884	Ua - Vd	0.998
C4 - Ci	0.196	Ci - Pl	0.998	Mp - Rs	0.999	Ua - Vl	1.000
C4 - Fo47	0.997	Ci - Rs	0.954	Mp - Ss	1.000	Vd - Vl	0.997
C4 - For	0.989	Ci - Ss	1.000	Mp - Tv	<.0001		
C4 - Mp	0.026	Ci - Tv	<.0001	Mp - Ua	0.063		
C4 - Pl	0.789	Ci - Ua	0.376	Mp - Vd	0.649		
C4 - Rs	0.002	Ci - Vd	0.970	Mp - Vl	0.049		
C4 - Ss	0.107	Ci - Vl	0.324	Pl - Rs	0.295		
C4 - Tv	<.0001	Fo47 - For	1.000	Pl - Ss	0.991		
C4 - Ua	1.000	Fo47 - Mp	0.380	Pl - Tv	<.0001		
C4 - Vd	0.971	Fo47 - Pl	1.000	Pl - Ua	0.949		
C4 - Vl	1.000	Fo47 - Rs	0.053	Pl - Vd	1.000		

(b) 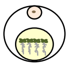

contrast	p.value	contrast	p.value	contrast	p.value
C10 - C4	1.000	C7 - Mp	0.463	For - Ss	0.533
C10 - C7	1.000	C7 - Pl	0.840	For - Tv	0.994
C10 - Ci	1.000	C7 - Ss	0.350	For - Ua	0.185
C10 - Fo47	0.979	C7 - Tv	0.001	For - Vd	0.020
C10 - For	0.014	C7 - Ua	0.787	For - Vl	0.151
C10 - Mp	0.976	C7 - Vd	0.998	Mp - Pl	1.000
C10 - Pl	0.998	C7 - Vl	0.657	Mp - Ss	1.000
C10 - Ss	0.894	Ci - Fo47	0.993	Mp - Tv	0.449
C10 - Tv	0.095	Ci - For	0.008	Mp - Ua	1.000
C10 - Ua	0.995	Ci - Mp	0.992	Mp - Vd	0.998
C10 - Vd	1.000	Ci - Pl	1.000	Mp - Vl	1.000
C10 - Vl	0.988	Ci - Ss	0.937	Pl - Ss	1.000
C4 - C7	1.000	Ci - Tv	0.064	Pl - Tv	0.416
C4 - Ci	0.999	Ci - Ua	0.999	Pl - Ua	1.000
C4 - Fo47	0.788	Ci - Vd	1.000	Pl - Vd	1.000
C4 - For	0.001	Ci - Vl	0.997	Pl - Vl	1.000
C4 - Mp	0.596	Fo47 - For	0.774	Ss - Tv	0.971
C4 - Pl	0.883	Fo47 - Mp	1.000	Ss - Ua	1.000
C4 - Ss	0.443	Fo47 - Pl	1.000	Ss - Vd	0.972
C4 - Tv	0.005	Fo47 - Ss	1.000	Ss - Vl	1.000
C4 - Ua	0.834	Fo47 - Tv	0.994	Tv - Ua	0.681
C4 - Vd	0.998	Fo47 - Ua	1.000	Tv - Vd	0.142
C4 - Vl	0.737	Fo47 - Vd	0.997	Tv - Vl	0.634
C7 - Ci	0.999	Fo47 - Vl	1.000	Ua - Vd	1.000
C7 - Fo47	0.760	For - Mp	0.078	Ua - Vl	1.000
C7 - For	0.000	For - Pl	0.077	Vd - Vl	0.999

Fig S5 Statistical output of the pairwise comparisons between the fungal volatile exposures in the absence (a) and presence (b) of *Arabidopsis thaliana* seedlings, using Tukey post-hoc tests.

Table S1 List of fungi with information on accession number, abbreviations used in this study, source, pathogenicity and incubation time

Fungus	Accession number	Abbreviations	Source	Pathogenicity	Incubation time
Chaetomium indicum	CBS 356.92	Ci	Westerdijk Fungal Biodiversity Institute (NL)	No	10 days
Fusarium oxysporum Fo47	Fo 47	Fo47	Agroécologie group - INRA Dijon (FR)	No	7 days
Fusarium oxysporum f.sp. raphani	WCS600	For	Microbe-Plant-Interactions group-Utrecht University (NL)	Yes	7 days
Mucor plumbeus R2	n/a	Mp	NIOO-KNAW collection (NL)	No	4 days
Phoma leveillei	CBS 373.69	Pl	Westerdijk Fungal Biodiversity Institute (NL)	No	10 days
Rhizoctonia solani AG-2-2 IIIB	n/a	Rs	Sugar Beet Research Institute (NL)	*Yes	7 days
Sclerotinia sclerotiorum	CBS 397.54	Ss	Westerdijk Fungal Biodiversity Institute (NL)	*Yes	10 days
Trichoderma viride	CBS 101227	Tv	Westerdijk Fungal Biodiversity Institute (NL)	No	4 days
Ulocladium atrum	CBS 193.67	Ua	Westerdijk Fungal Biodiversity Institute (NL)	No	7 days
Verticillium dahliae JR2	n/a	Vd	Phytopathology group Wageningen University (NL)	Yes	7 days
Verticillium longisporum	CBS 110219	Vl	Westerdijk Fungal Biodiversity Institute (NL)	*Yes	7 days

The pathogenicity reported for these fungi is based on the literature available for brassicaceous species. The strains for which the pathogenicity was not specifically confirmed in *Arabidopsis thaliana* Col-0 are marked with "*Yes" in the pathogenicity and were tested *via* a pathogenicity assay in this study.

Table S2 List of fungal pathogens used in this study and their corresponding host range specificity, disease symptoms and infection mechanisms

Fungal species	Host-range	Disease	Infection mechanism	Symptoms in Arabidopsis	References
Fusarium oxysporum f. sp. raphani	Radish and Arabidopsis	Yellow/wilt	Through root tips, wounds in the roots, or at the formation point of lateral roots	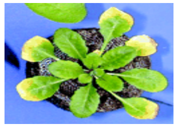	(Bosland&Williams, 1987, Diener&Ausubel, 2005)
Rhizoctonia solani	Broad, including bean, lettuce, sugar beet, and cabbage	Bottom Rot/ Damping-off	Through direct penetration of the plant cuticle/epidermis or by means of natural openings in the plant	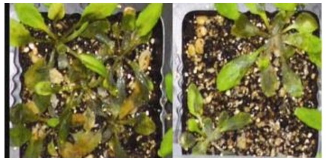	(Perl-Treves *et al.*, 2004, Sneh *et al.*, 1986); (Foley *et al.*, 2013)
Sclerotinia sclerotiorum	Broad (more than 400 species) including Arabidopsis	Stem rot and watery soft rot	Through flowers or by direct germination on leaves (arpogenic germination) or through roots, crowns and lower plant parts (myceliogenic germination)	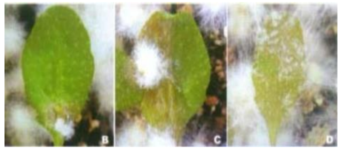	(Li *et al.*, 2003); (Dixelius *et al.*, 2004)
Verticillium dahliae	Broad, including eggplant, cabbage, and tomato	Wilt	Through lateral roots and root hairs	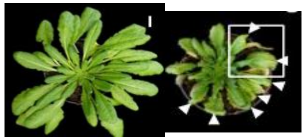	(Fradin&Thomma, 2006, Tjamos *et al.*, 2005); (Zhou *et al.*, 2006)
Verticillium longisporum	Brassica crops	Wilt	Through lateral roots and root hairs	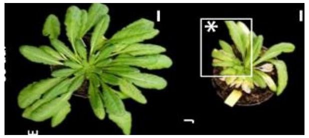	(Karapapa *et al.*, 1997, Zhou *et al.*, 2006)

Table S3 Volatile organic compounds (mean peak area 10^{-6} mg fungal biomass^{-1} ± SD) quantified in the headspace of the pathogenic fungi and non-pathogenic fungi using total ion chromatograms

	Pathogenic fungi					Non-pathogenic fungi					
	For	*Rs*	*Ss*	*Vd*	*Vl*	*Ci*	*Fo 47*	*Mp*	*Pl*	*Tv*	*Ua*
**Amino acid and fatty acid derivates**											
1,3-butadiene, 2-methyl	ND	ND	ND	ND	ND	951 ± 231	ND	ND	ND	ND	ND
1,5-octadien-3-ol, (*Z*)	ND	10 ± 4	ND	ND	ND	ND	ND	ND	ND	ND	ND
1-butanol	ND	ND	ND	ND	ND	47 ± 15	ND	ND	ND	ND	ND
1-decanol acetate	12 ± 11	ND	ND	ND	ND	ND	ND	ND	ND	ND	ND
1-hexanol	ND	ND	ND	ND	ND	ND	207 ± 185	ND	ND	ND	ND
1-octen-3-ol	ND	405 ± 119	21 ± 15	BT (3.91)	16 ± 5	91 ± 50	ND	ND	ND	27 ± 14	5 ± 3
1-octen-3-one	ND	17 ± 7	ND	ND	ND	5 ± 1	ND	ND	ND	26 ± 32	ND
2,3-butanedione	230 ± 170	ND	ND	ND	ND	ND	BT (3.21)	199 ± 58	ND	ND	BT (2.15)
2,4-hexadienal, (*E*,*E*)	ND	ND	ND	ND	ND	8 ± 5	ND	ND	ND	ND	ND
2-butanone	ND	ND	56 ± 39	ND	ND	106 ± 59	ND	411 ± 67	ND	ND	ND

Ci: *Chaetomium indicum*, Fo47: *Fusarium oxysporum* 47, For: *F. oxysporum* f.sp. *raphani*, Mp: *Mucor plumbeus*, Pl: *Phoma leveillei*, Rs: *Rhizoctonia solani*, Ss: *Sclerotinia sclerotiorum*, Tv: *Trichoderma viride*, Ua: *Ulocladium atrum*, Vd: *Verticillium dahliae*, Vl: *Verticillium longisporum*. "ND" indicates that the compound was not detected in the samples or in less than 50% of the samples of the fungus; "BT" indicates that the compound was detected but the peak area was below the selected threshold of 4 (emission 4-fold higher than controls). Ratio of peak area measured in the sample over the controls is reported between brackets in the latter case.

Table S3 ***Continued***

	Pathogenic fungi					Non-pathogenic fungi					
	For	*Rs*	*Ss*	*Vd*	*Vl*	*Ci*	*Fo 47*	*Mp*	*Pl*	*Tv*	*Ua*
**Amino acid and fatty acid derivates *(continued)***											
2-butenal, 2-methyl-,(*E*)	ND	ND	ND	ND	ND	2 ± 1	ND	ND	ND	ND	ND
2-butenal, 3-methyl	21 ± 15	ND	ND	ND	ND	279 ± 58	ND	15 ± 5	ND	ND	ND
2-heptanone	BT (1.61)	ND	ND	4 ± 1	ND	ND	ND	ND	ND	ND	ND
2-methyl-1-butyl acetate	9 ± 14	ND	ND	ND	ND	ND	ND	ND	ND	ND	ND
2-methyl-1-propanol	2056 ± 885	ND	ND	ND	ND	ND	621 ± 644	449 ± 115	61 ± 8	111± 37	156 ± 75
2-ethyl-3-buten-2-ol	ND	ND	ND	ND	ND	948 ± 136	ND	ND	ND	ND	ND
2-methyl propanal	81 ± 885	ND	ND	ND	ND	ND	BT (2.09)	ND	ND	ND	ND
2-nonen-4-one, (*E*)	ND	ND	ND	ND	ND	ND	ND	ND	ND	87 ± 85	ND
2-pentylfuran	ND	ND	ND	ND	ND	ND	ND	ND	ND	43 ± 12	ND
2-undecanone	ND	ND	ND	25 ± 11	ND	ND	ND	ND	ND	ND	ND
3-heptanone, 4-methyl	ND	ND	11 ± 2	ND	ND	ND	ND	ND	ND	ND	ND

Ci: *Chaetomium indicum*, Fo47: *Fusarium oxysporum* 47, For: *F. oxysporum* f.sp. *raphani*, Mp: *Mucor plumbeus*, Pl: *Phoma leveillei*, Rs: *Rhizoctonia solani*, Ss: *Sclerotinia sclerotiorum*, Tv: *Trichoderma viride*, Ua: *Ulocladium atrum*, Vd: *Verticillium dahliae*, Vl: *Verticillium longisporum*. "ND" indicates that the compound was not detected in the samples or in less than 50% of the samples of the fungus; "BT" indicates that the compound was detected but the peak area was below the threshold of 4 (emission 4-fold higher than controls). Ratio of peak area measured in the sample over the controls is reported between brackets in the latter case.

Table S3 ***Continued***

	Pathogenic fungi					Non-pathogenic fungi					
	For	*Rs*	*Ss*	*Vd*	*Vl*	*Ci*	*Fo 47*	*Mp*	*Pl*	*Tv*	*Ua*
**Amino acid and fatty acid derivates *(continued)***											
3-heptanone, 5-methyl	ND	ND	ND	ND	ND	6 ± 2	ND	ND	ND	46 ± 26	ND
3-hydroxy-2-butanone	195 ± 141	ND	ND	ND	ND	ND	ND	612 ± 246	ND	ND	8 ± 9
3-methyl-1-butanol and/or 2-methyl-1-butanol and/or 1-pentanol*	7434 ± 3342	ND	359 ± 138	1747 ± 1907	131 ± 66	85 ± 28	1102 ± 1336	4440 ± 522	103 ± 34	272 ± 140	200 ± 64
3-methyl-1-butanol, acetate	26 ± 33	ND	ND	ND	ND	ND	ND	ND	ND	ND	ND
3-methyl-2-buten-1-ol	ND	ND	ND	ND	ND	309 ± 163	ND	ND	ND	ND	ND
3-methyl-2-pentanone	ND	ND	ND	ND	ND	36 ± 25	ND	ND	ND	ND	ND
3-methyl-3-buten-1-ol	33 ± 18	ND	10± 4	24 ± 12	25 ± 11	113 ± 35	21± 23	ND	7± 4	ND	ND
3-methyl butanal, oxime, anti	ND	ND	ND	ND	ND	6 ± 1	ND	ND	ND	ND	ND

Ci: *Chaetomium indicum*, Fo47: *Fusarium oxysporum* 47, For: *F. oxysporum* f.sp. *raphani*, Mp: *Mucor plumbeus*, Pl: *Phoma leveillei*, Rs: *Rhizoctonia solani*, Ss: *Sclerotinia sclerotiorum*, Tv: *Trichoderma viride*, Ua: *Ulocladium atrum*, Vd: *Verticillium dahliae*, Vl: *Verticillium longisporum*. "ND" indicates that the compound was not detected in the samples or in less than 50% of the samples of the fungus; "BT" indicates that the compound was detected but the peak area was below the threshold of 4 (emission 4-fold higher than controls). "*" indicates the presence of 3-methyl-1-butanol and/or 2-methyl-1-butanol and/or 1-pentanol due to a high co-elution.

Table S3 ***Continued***

	Pathogenic fungi					Non-pathogenic fungi					
	For	*Rs*	*Ss*	*Vd*	*Vl*	*Ci*	*Fo 47*	*Mp*	*Pl*	*Tv*	*Ua*
**Amino acid and fatty acid derivates *(continued)***											
3-octanol	ND	ND	ND	26± 33	11 ± 6	ND	ND	ND	ND	ND	ND
3-octanol acetate	ND	ND	ND	ND	ND	6 ± 2	ND	ND	ND	ND	ND
3-octanone	ND	66 ± 23	ND	150 ± 80	ND	ND	ND	ND	ND	ND	ND
4-heptanol	1951 ± 1740	ND	1262 ± 2094	ND	411 ± 375	ND	1270 ± 1239	635 ± 730	963 ± 721	893 ± 830	777 ± 955
4-methyl-2-pentanone	68 ± 59	ND	ND	ND	ND	ND	BT (1.74)	ND	3± 2	ND	ND
6-pentyl-2H-pyran-2-one	ND	ND	ND	ND	ND	ND	ND	16 ± 17	ND	2023 ± 575	16 ± 12
allyl hexanoate	ND	ND	ND	ND	ND	ND	ND	ND	ND	13 ± 12	ND
cyclohexanone	17 ± 17	ND	ND	ND	ND	ND	ND	ND	ND	ND	ND
ethyl acetate	190 ± 251	ND	ND	ND	ND	ND	BT (2.24)	ND	ND	ND	ND
ethyl tiglate	ND	ND	ND	ND	ND	ND	12 ± 7	ND	ND	ND	ND
furfuryl alcohol	ND	ND	ND	ND	ND	ND	ND	22± 6	ND	ND	ND

Ci: *Chaetomium indicum*, Fo47: *Fusarium oxysporum* 47, For: *F. oxysporum* f.sp. *raphani*, Mp: *Mucor plumbeus*, Pl: *Phoma leveillei*, Rs: *Rhizoctonia solani*, Ss: *Sclerotinia sclerotiorum*, Tv: *Trichoderma viride*, Ua: *Ulocladium atrum*, Vd: *Verticillium dahliae*, Vl: *Verticillium longisporum*. "ND" indicates that the compound was not detected in the samples or in less than 50% of the samples of the fungus; "BT" indicates that the compound was detected but the peak area was below the threshold of 4 (emission 4-fold higher than controls). Ratio of peak area measured in the sample over the controls is reported between brackets in the latter case.

Table S3 ***Continued***

	Pathogenic fungi					Non-pathogenic fungi					
	For	*Rs*	*Ss*	*Vd*	*Vl*	*Ci*	*Fo 47*	*Mp*	*Pl*	*Tv*	*Ua*
**Amino acid and fatty acid derivates** ***(continued)***											
γ-decalactone	ND	ND	ND	7 ± 4	ND	ND	ND	ND	ND	ND	ND
methyl tiglate	20± 29	ND	ND	ND	ND	ND	ND	ND	ND	ND	ND
nonyl acetate	14 ± 12	ND	ND	ND	ND	ND	ND	ND	ND	ND	ND
prenyl formate	ND	ND	ND	ND	ND	ND	343 ± 160	ND	ND	ND	ND
**Benzenoids**											
2-phenyl ethanol	ND	ND	ND	196 ± 168	67± 19	ND	ND	172 ± 35	ND	ND	BT (1.40)
anisole, p-ethyl	ND	ND	ND	ND	ND	ND	123 ± 84	ND	ND	ND	ND
anisole, p-vinyl	ND	ND	ND	ND	ND	ND	22 ± 12	ND	ND	ND	ND
benzyl alcohol	ND	ND	ND	ND	ND	5 ± 2	ND	ND	ND	ND	ND
phenol	ND	ND	ND	ND	ND	18 ± 8	ND	ND	ND	BT (1.93)	ND
p-methyl acetophenone	ND	ND	ND	ND	ND	ND	94 ± 39	ND	ND	ND	ND

Ci: *Chaetomium indicum*, Fo47: *Fusarium oxysporum* 47, For*: F. oxysporum* f.sp. *raphani*, Mp: *Mucor plumbeus*, Pl: *Phoma leveillei*, Rs: *Rhizoctonia solani*, Ss*: Sclerotinia sclerotiorum*, Tv: *Trichoderma viride*, Ua: *Ulocladium atrum*, Vd: *Verticillium dahliae*, Vl: *Verticillium longisporum*. "ND" indicates that the compound was not detected in the samples or in less than 50% of the samples of the fungus; "BT" indicates that the compound was detected but the peak area was below the threshold of 4 (emission 4-fold higher than controls). Ratio of peak area measured in the sample over the controls is reported between brackets in the latter case.

Table S3 ***Continued***

	Pathogenic fungi					Non-pathogenic fungi					
	For	*Rs*	*Ss*	*Vd*	*Vl*	*Ci*	*Fo 47*	*Mp*	*Pl*	*Tv*	*Ua*
**Benzenoids *(continued)***											
styrene	ND	ND	ND	ND	ND	ND	798 ± 172	ND	ND	ND	ND
**Monoterpenoids**											
linalool	ND	13 ± 9	40 ± 20	ND	ND	ND	ND	ND	ND	ND	ND
linalool oxide B	ND	ND	16± 6	ND	ND	ND	ND	ND	ND	ND	ND
phellandral	ND	ND	ND	ND	ND	18 ± 5	ND	ND	ND	ND	ND
terpinolene	ND	ND	ND	ND	ND	ND	16 ± 8	ND	ND	ND	ND
**Sesquiterpenes**											
2-epi-β-caryophyllene, (*E*)	24 ± 20	ND	ND	ND	ND	ND	ND	ND	ND	ND	ND
alloaromadendrene	33 ± 21	ND	ND	ND	ND	ND	ND	ND	ND	ND	ND
α-zingiberene	ND	ND	ND	ND	ND	ND	ND	ND	ND	38 ± 15	ND

Ci: *Chaetomium indicum*, Fo47: *Fusarium oxysporum* 47, For: *F. oxysporum* f.sp. *raphani*, Mp: *Mucor plumbeus*, Pl: *Phoma leveillei*, Rs: *Rhizoctonia solani*, Ss: *Sclerotinia sclerotiorum*, Tv: *Trichoderma viride*, Ua: *Ulocladium atrum*, Vd: *Verticillium dahliae*, Vl: *Verticillium longisporum*. "ND" indicates that the compound was not detected in the samples or in less than 50% of the samples of the fungus; "BT" indicates that the compound was detected but the peak area was below the threshold of 4 (emission 4-fold higher than controls). Ratio of peak area measured in the sample over the controls is reported between brackets in the latter case.

Table S3 ***Continued***

	Pathogenic fungi					Non-pathogenic fungi					
	For	*Rs*	*Ss*	*Vd*	*Vl*	*Ci*	*Fo 47*	*Mp*	*Pl*	*Tv*	*Ua*
**Sesquiterpenes *(continued)***											
aromadendr-9-ene	215 ± 139	ND	ND	ND	ND	ND	ND	ND	ND	ND	ND
β-caryophyllene, (*E*)	8± 5	ND	ND	ND	ND	ND	ND	ND	ND	ND	ND
β-sesqui phellandrene	ND	ND	ND	ND	ND	ND	ND	ND	ND	60± 28	ND
caryophyllene oxide	ND	ND	ND	ND	ND	ND	ND	ND	ND	62 ± 26	ND
γ-elemene	ND	ND	ND	ND	ND	ND	13 ± 5	ND	ND	ND	ND
γ-patchoulene	ND	ND	ND	ND	ND	ND	145 ± 100	ND	ND	ND	ND
nerolidol, (*E*)	ND	5 ± 3	ND	ND	ND	ND	ND	ND	ND	ND	ND
nerolidol, (*Z*)	ND	ND	ND	ND	ND	ND	ND	ND	ND	31 ± 22	ND
sesquisabinene hydrate, (*E*)	ND	ND	ND	ND	ND	ND	ND	ND	ND	31 ± 18	ND

Ci: *Chaetomium indicum*, Fo47: *Fusarium oxysporum* 47, For*: F. oxysporum* f.sp. *raphani*, Mp: *Mucor plumbeus*, Pl: *Phoma leveillei*, Rs: *Rhizoctonia solani*, Ss*: Sclerotinia sclerotiorum*, Tv: *Trichoderma viride*, Ua: *Ulocladium atrum*, Vd: *Verticillium dahliae*, Vl: *Verticillium longisporum*. "ND" indicates that the compound was not detected in the samples or in less than 50% of the samples of the fungus; "BT" indicate that the compound was detected but the peak area was below the threshold of 4 (emission 4-fold higher than controls). Ratio of peak area measured in the sample over the controls are reported between brackets in the latter case.

Table S3 ***Continued***

	Pathogenic fungi					Non-pathogenic fungi					
	For	*Rs*	*Ss*	*Vd*	*Vl*	*Ci*	*Fo 47*	*Mp*	*Pl*	*Tv*	*Ua*
**N-containing compounds**											
benzoic acid, 2-(methylamino), methyl ester	ND	ND	ND	ND	ND	ND	ND	ND	ND	77 ± 91	ND
**S-containing compounds**											
methyl thiocyanate	ND	27 ± 8	ND	ND	ND	ND	ND	ND	ND	ND	ND
**Unknown compounds**											
unknown m/z 100	ND	ND	ND	ND	ND	19 ± 5	ND	ND	ND	ND	ND
unknown m/z 113	ND	ND	ND	BT (1.40)	ND	6 ± 1	ND	ND	ND	ND	ND
unknown m/z 114	ND	ND	ND	ND	ND	4 ± 1	ND	ND	ND	ND	ND
unknown m/z 120	ND	ND	ND	59 ± 95	ND	ND	ND	ND	ND	ND	ND
unknown m/z 129	ND	22 ± 24	25 ± 26	36 ± 29	ND	ND	ND	ND	ND	ND	ND

Ci: *Chaetomium indicum*, Fo47: *Fusarium oxysporum* 47, For: *F. oxysporum* f.sp. *raphani*, Mp: *Mucor plumbeus*, Pl: *Phoma leveillei*, Rs: *Rhizoctonia solani*, Ss: *Sclerotinia sclerotiorum*, Tv: *Trichoderma viride*, Ua: *Ulocladium atrum*, Vd: *Verticillium dahliae*, Vl: *Verticillium longisporum*. "ND" indicates that the compound was not detected in the samples or in less than 50% of the samples of the fungus; "BT" indicate that the compound was detected but the peak area was below the threshold of 4 (emission 4-fold higher than controls). Ratio of peak area measured in the sample over the controls are reported between brackets in the latter case.

Table S3 ***Continued***

	Pathogenic fungi					Non-pathogenic fungi					
	For	*Rs*	*Ss*	*Vd*	*Vl*	*Ci*	*Fo 47*	*Mp*	*Pl*	*Tv*	*Ua*
**Unknown compounds *(continued)***											
unknown m/z 138	ND	ND	ND	ND	ND	ND	BT (1.11)	ND	ND	34 ± 49	ND
unknown m/z 146	ND	ND	ND	ND	ND	19 ± 5	ND	ND	ND	ND	ND
unknown m/z 149	25 ± 20	ND	ND	ND	ND	ND	ND	ND	ND	ND	ND
unknown m/z 161	1003± 714	ND	ND	ND	ND	ND	ND	ND	ND	ND	ND
unknown m/z 164.12	110 ± 79	ND	ND	ND	ND	ND	ND	ND	ND	ND	ND
unknown m/z 164.22	23 ± 15	ND	ND	ND	ND	ND	ND	ND	ND	ND	ND
unknown m/z 189	13 ± 14	ND	ND	ND	ND	ND	ND	ND	ND	ND	ND
unknown m/z 73	ND	4 ± 4	ND	ND	ND	ND	ND	ND	ND	ND	ND
**TOTAL VOCs**	**12848 ± 6110**	**572 ± 159**	**1380 ± 1886**	**2277 ± 2187**	**526 ± 334**	**3097 ± 609**	**4055 ± 2469**	**6977 ± 909**	**818 ± 782**	**3584 ± 1708**	**1027 ± 868**

Ci: Chaetomium indicum, Fo47: *Fusarium oxysporum* 47, For*: F. oxysporum* f.sp. *raphani*, Mp: *Mucor plumbeus*, Pl: *Phoma leveillei*, Rs: *Rhizoctonia solani*, Ss*: Sclerotinia sclerotiorum*, Tv: *Trichoderma viride*, Ua: *Ulocladium atrum*, Vd: *Verticillium dahliae*, Vl: *Verticillium longisporum*. "ND" indicates that the compound was not detected in the samples or in less than 50% of the samples of the fungus; "BT" indicates that the compound was detected but the peak area was below the threshold of 4 (emission 4-fold higher than controls). Ratio of peak area measured in the sample over the controls is reported between brackets in the latter case.

Table S4 List of the variable importance in projection (VIP) values, generated by the PLS-DA model, for each volatile organic compound of the fungal headspace

	VIP		VIP
unknown m/z 129	1.73247	2-butenal, 3-methyl	0.980837
linalool	1.58972	unknown m/z 73	0.976175
3-octanone	1.53772	2-methylpropanal	0.972724
linalool oxide B	1.34451	4-methyl-2-pentanone	0.967578
3-heptanone, 4-methyl	1.28484	γ-elemene	0.961433
furfuryl alcohol	1.28081	3-heptanone, 5-methyl	0.960106
methyl thiocyanate	1.25267	3-methyl-1-butanol, acetate	0.955109
2-butanone	1.21264	perilla ketone	0.950891
1-octen-3-ol	1.20929	3-methyl-2-buten-1-ol	0.950432
1,5-octadien-3-ol, (*Z*)	1.20690	ethyl acetate	0.945610
2-heptanone	1.20230	2,3-butanedione	0.938122
2-undecanone	1.15551	2-pentylfuran	0.935906
3-octanol	1.13733	co-elution compounds	0.933692
3-hydroxy-2-butanone	1.08861	methyl tiglate	0.931901
nerolidol, (*E*)	1.06419	p-methylacetophenone	0.929315
unknown m/z 164.22	1.06124	2,4-hexadienal, (*E*,*E*)	0.925762
2-methyl-3-buten-2-ol	1.05348	3-methyl-2-pentanone	0.925220
1,3-butadiene, 2-methyl	1.04689	prenyl formate	0.919730
β-caryophyllene, (*E*)	1.04492	2-methyl-1-butyl acetate	0.918724
unknown m/z 100	1.03726	terpinolene	0.912356
unknown m/z 161	1.03495	caryophyllene oxide	0.896991
unknown m/z 149	1.03401	α-zingiberene	0.895012
γ-decalactone	1.02718	anisole, p-vinyl	0.890064
unknown m/z 164.12	1.02491	β-sesquiphellandrene	0.881373
1-butanol	1.02276	ethyl tiglate	0.863652
phellandral	1.02154	γ-patchoulene	0.857320
unknown m/z 113	1.02145	unknown m/z 189	0.848463
aromadendr-9-ene	1.02043	anisole, p-ethyl	0.837471
3-octanol acetate	1.02026	sesquisabinene hydrate, (*E*)	0.836242
2-epi-β-caryophyllene, (*E*)	1.01620	2-methyl-1-propanol	0.814313
3-methylbutanal, oxime, anti	1.01537	nerolidol, (*Z*)	0.790710
nonyl acetate	1.01447	3-methyl-3-buten-1-ol	0.751673
unknown m/z 146	1.01429	allyl hexanoate	0.723355
alloaromadendrene	1.01412	2-nonen-4-one, (*E*)	0.701143
2-butenal, 2-methyl-,(*E*)	1.01339	unknown m/z 120	0.695870
1-decanol acetate	1.01139	benzoic acid, 2-(methylamino)	0.636713
benzyl alcohol	1.00526	unknown m/z 138	0.563735
unknown m/z 114	0.998043	1-hexanol	0.544389
cyclohexanone	0.995420	2-phenylethanol	0.460180
styrene	0.993565	1-octen-3-one	0.413256
phenol	0.984344	4-heptanol	0.399529

References of the Electronic Supplemental Material

Bosland P, W., Williams PH. 1987. An evaluation of *Fusarium oxysporum* from crucifers based on pathogenicity, isozyme polymorphism, vegetative compatibility, and geographic origin. *Can J Bot* **65**: 2067-2073 doi: 10.1139/b87-282

Diener AC, Ausubel FM. 2005. Resistance to *Fusarium oxysporum* 1, a dominant Arabidopsis disease-resistance gene, is not race specific. *Genetics* **171**: 305-321 doi: 10.1534/genetics.105.042218

Dixelius C, Bohman S, Wretblad S. 2004. Disease resistance. In: *Brassica*. Berlin, Heidelberg: Springer, p. 253-271.

Foley RC, Gleason CA, Anderson JP, Hamann T, Singh KB. 2013. Genetic and genomic analysis of *Rhizoctonia solani* interactions with Arabidopsis; Evidence of resistance mediated through NADPH oxidases. *Plos One* **8**: e56814 doi: 10.1371/journal.pone.0056814

Fradin EF, Thomma BPHJ. 2006. Physiology and molecular aspects of *Verticillium* wilt diseases caused by *V. dahliae* and *V. albo-atrum*. *Mol Plant Pathol* **7**: 71-86 doi: 10.1111/j.1364-3703.2006.00323.x

Hoagland DR, Arnon DI (1950) The water-culture method for growing plants without soil. C*ircular California Agri Exp Station* 347

Karapapa VK, Bainbridge BW, Heale JB. 1997. Morphological and molecular characterization of *Verticillium longisporum* comb, nov., pathogenic to oilseed rape. *Mycol Res* **101**: 1281-1294 doi: 10.1017/S0953756297003985

Li GQ, Huang HC, Acharya SN. 2003. Antagonism and biocontrol potential of *Ulocladium atrum* on *Sclerotinia sclerotiorum*. *Biol Cont* **28**: 11-18 doi: 10.1016/S1049-9644(03)00050-1

Perl-Treves R, Foley RC, Chen W, Singh KB. 2004. Early induction of the Arabidopsis GSTF8 promoter by specific strains of the fungal pathogen *Rhizoctonia solani*. *Mol Plant-Microbe Interac* **17**: 70-80 doi: 10.1094/MPMI.2004.17.1.70

Sneh B, Zeidan M, Ichielevich-Auster M, Barash I, Koltin Y. 1986. Increased growth responses induced by a nonpathogenic *Rhizoctonia solani*. *Can J Bot* **64**: 2372-2378 doi: 10.1139/b86-313

Tjamos SE, Flemetakis E, Paplomatas EJ, Katinakis P. 2005. Induction of resistance to *Verticillium dahliae* in *Arabidopsis thaliana* by the biocontrol agent K-165 and pathogenesis-related proteins gene expression. *Mol Plant-Microbe Interac* **18**: 555-561 doi: 10.1094/MPMI-18-0555

Zhou L, Hu Q, Johansson A, Dixelius C. 2006. *Verticillium longisporum* and *V. dahliae*: infection and disease in *Brassica napus*. *Plant Pathol* **55**: 137-144 doi: 10.1111/j.1365-3059.2005.01311.x
